# Multielectrode catheter-based pulsed electric field vs. cryoballoon for atrial fibrillation ablation: a systematic review and meta-analysis

**DOI:** 10.1093/europace/euae293

**Published:** 2024-11-23

**Authors:** Giampaolo Vetta, Domenico Giovanni Della Rocca, Antonio Parlavecchio, Michele Magnocavallo, Antonio Sorgente, Luigi Pannone, Alvise Del Monte, Alexandre Almorad, Juan Sieira, Lorenzo Marcon, Ioannis Doundoulakis, Sanghamitra Mohanty, Charles Audiat, Kazutaka Nakasone, Gezim Bala, Erwin Ströker, Stéphane Combes, Ingrid Overeinder, Stefano Bianchi, Pietro Palmisano, Pietro Rossi, Serge Boveda, Marc La Meir, Andrea Natale, Andrea Sarkozy, Carlo de Asmundis, Gian-Battista Chierchia

**Affiliations:** Heart Rhythm Management Centre, Postgraduate Program in Cardiac Electrophysiology and Pacing, Universitair Ziekenhuis Brussel-Vrije Universiteit Brussel, European Reference Networks Guard-Heart, Brussels 1090, Belgium; Mediterranean Consortium for Arrhythmia Research (MediCAR), Rome, Italy; Heart Rhythm Management Centre, Postgraduate Program in Cardiac Electrophysiology and Pacing, Universitair Ziekenhuis Brussel-Vrije Universiteit Brussel, European Reference Networks Guard-Heart, Brussels 1090, Belgium; Mediterranean Consortium for Arrhythmia Research (MediCAR), Rome, Italy; Texas Cardiac Arrhythmia Institute, St. David's Medical Center, Austin, TX 78705, USA; Department of Clinical and Experimental Medicine, Cardiology Unit, University of Messina, Messina, Italy; Mediterranean Consortium for Arrhythmia Research (MediCAR), Rome, Italy; Arrhythmology Unit, Ospedale Fatebenefratelli Isola Tiberina-Gemelli Isola, Rome, Italy; Heart Rhythm Management Centre, Postgraduate Program in Cardiac Electrophysiology and Pacing, Universitair Ziekenhuis Brussel-Vrije Universiteit Brussel, European Reference Networks Guard-Heart, Brussels 1090, Belgium; Heart Rhythm Management Centre, Postgraduate Program in Cardiac Electrophysiology and Pacing, Universitair Ziekenhuis Brussel-Vrije Universiteit Brussel, European Reference Networks Guard-Heart, Brussels 1090, Belgium; Heart Rhythm Management Centre, Postgraduate Program in Cardiac Electrophysiology and Pacing, Universitair Ziekenhuis Brussel-Vrije Universiteit Brussel, European Reference Networks Guard-Heart, Brussels 1090, Belgium; Heart Rhythm Management Centre, Postgraduate Program in Cardiac Electrophysiology and Pacing, Universitair Ziekenhuis Brussel-Vrije Universiteit Brussel, European Reference Networks Guard-Heart, Brussels 1090, Belgium; Heart Rhythm Management Centre, Postgraduate Program in Cardiac Electrophysiology and Pacing, Universitair Ziekenhuis Brussel-Vrije Universiteit Brussel, European Reference Networks Guard-Heart, Brussels 1090, Belgium; Heart Rhythm Management Centre, Postgraduate Program in Cardiac Electrophysiology and Pacing, Universitair Ziekenhuis Brussel-Vrije Universiteit Brussel, European Reference Networks Guard-Heart, Brussels 1090, Belgium; Heart Rhythm Management Centre, Postgraduate Program in Cardiac Electrophysiology and Pacing, Universitair Ziekenhuis Brussel-Vrije Universiteit Brussel, European Reference Networks Guard-Heart, Brussels 1090, Belgium; Texas Cardiac Arrhythmia Institute, St. David's Medical Center, Austin, TX 78705, USA; Heart Rhythm Management Centre, Postgraduate Program in Cardiac Electrophysiology and Pacing, Universitair Ziekenhuis Brussel-Vrije Universiteit Brussel, European Reference Networks Guard-Heart, Brussels 1090, Belgium; Heart Rhythm Management Centre, Postgraduate Program in Cardiac Electrophysiology and Pacing, Universitair Ziekenhuis Brussel-Vrije Universiteit Brussel, European Reference Networks Guard-Heart, Brussels 1090, Belgium; Heart Rhythm Management Centre, Postgraduate Program in Cardiac Electrophysiology and Pacing, Universitair Ziekenhuis Brussel-Vrije Universiteit Brussel, European Reference Networks Guard-Heart, Brussels 1090, Belgium; Heart Rhythm Management Centre, Postgraduate Program in Cardiac Electrophysiology and Pacing, Universitair Ziekenhuis Brussel-Vrije Universiteit Brussel, European Reference Networks Guard-Heart, Brussels 1090, Belgium; Heart Rhythm Department, Clinique Pasteur, Toulouse, France; Heart Rhythm Management Centre, Postgraduate Program in Cardiac Electrophysiology and Pacing, Universitair Ziekenhuis Brussel-Vrije Universiteit Brussel, European Reference Networks Guard-Heart, Brussels 1090, Belgium; Arrhythmology Unit, Ospedale Fatebenefratelli Isola Tiberina-Gemelli Isola, Rome, Italy; Cardiology Unit, ‘Card. G. Panico’ Hospital, Tricase, Italy; Arrhythmology Unit, Ospedale Fatebenefratelli Isola Tiberina-Gemelli Isola, Rome, Italy; Heart Rhythm Department, Clinique Pasteur, Toulouse, France; Heart Rhythm Management Centre, Postgraduate Program in Cardiac Electrophysiology and Pacing, Universitair Ziekenhuis Brussel-Vrije Universiteit Brussel, European Reference Networks Guard-Heart, Brussels 1090, Belgium; Texas Cardiac Arrhythmia Institute, St. David's Medical Center, Austin, TX 78705, USA; Heart Rhythm Management Centre, Postgraduate Program in Cardiac Electrophysiology and Pacing, Universitair Ziekenhuis Brussel-Vrije Universiteit Brussel, European Reference Networks Guard-Heart, Brussels 1090, Belgium; Heart Rhythm Management Centre, Postgraduate Program in Cardiac Electrophysiology and Pacing, Universitair Ziekenhuis Brussel-Vrije Universiteit Brussel, European Reference Networks Guard-Heart, Brussels 1090, Belgium; Heart Rhythm Management Centre, Postgraduate Program in Cardiac Electrophysiology and Pacing, Universitair Ziekenhuis Brussel-Vrije Universiteit Brussel, European Reference Networks Guard-Heart, Brussels 1090, Belgium

**Keywords:** Atrial fibrillation, Ablation, Pulmonary vein isolation, Pulsed field ablation, Cryoballoon ablation, Catheter ablation

## Abstract

**Aims:**

Pulsed field ablation (PFA) is an innovative technology recently adopted for the treatment of atrial fibrillation (AF). Preclinical and clinical studies have reported a remarkable safety profile, as a result of its tissue-specific effect targeting cardiomyocytes and sparing adjacent tissues. Single-shot pentaspline system was the first PFA device to receive regulatory approval. We performed a meta-analysis to compare the efficacy and safety of PFA with the single-shot pentaspline system vs. currently available second-/third-/fourth-generation cryoballoon ablation (CRYO) technologies.

**Methods and results:**

We systematically searched electronic databases for studies focusing on AF ablation employing the PFA single-shot pentaspline system or second-/third-/fourth-generation CRYO technologies. The primary endpoints were acute procedural success assessed on a vein and patient basis. Safety endpoints included overall periprocedural complications and major periprocedural complications. We also compared procedural, fluoroscopy times, and freedom from atrial tachyarrhythmias (ATs) at follow-up (secondary endpoints). Twenty and 70 studies were included for PFA and CRYO, respectively. Pulsed field ablation demonstrated greater acute procedural success on a vein basis (99.9% vs. 99.1%; *P* < 0.001), as well as per patient (99.5% vs. 98.4%; *P* < 0.001). Pulsed field ablation yielded lower overall periprocedural complications (3.1% vs. 5.6%; *P* < 0.001), shorter procedural time (75.9 min vs. 105.6 min; *P* < 0.001), and fluoroscopy time (14.2 min vs. 18.9 min; *P* < 0.001) compared with CRYO. No differences were found for major periprocedural complications (1.2% vs. 1.0%; *P* = 0.46) and freedom from ATs at 1 year (82.3% vs. 80.3%; log-rank *P* = 0.61).

**Conclusion:**

Pulsed field ablation contributed to higher acute procedural success and safety compared with CRYO. No statistically significant differences in AT recurrence at 1-year follow-up were observed.

## Graphical Abstract

**Graphical Abstract:**
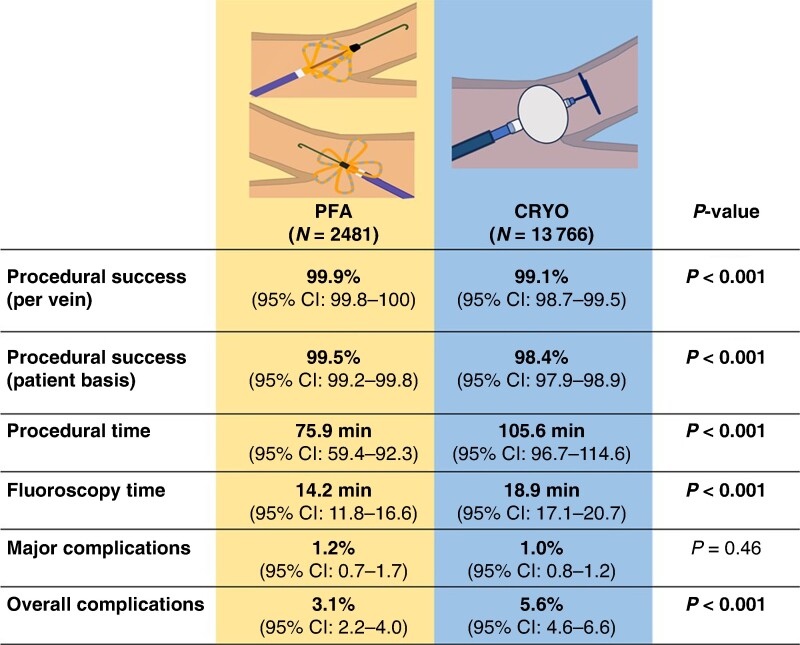
Efficacy and safety of pulse field ablation and cryoballoon ablation for atrial fibrillation ablation. CRYO, cryoballoon ablation; PFA, pulsed field ablation.

**What’s new?**

- Pulsed field ablation via the pentaspline catheter contributed to greater acute procedural success, assessed either on a pulmonary vein and a patient basis, compared with cryoballoon.
- Pulsed field ablation-based procedures showed a shorter procedural time and X-ray exposure.
- Pulsed electric field demonstrated a remarkable safety profile, as demonstrated by a significantly lower incidence of overall complications.
- Freedom from atrial tachyarrhythmias at 1 year was high (>80.0%) and similar between technologies.

## Introduction

Catheter ablation (CA) is a highly effective rhythm control strategy for paroxysmal atrial fibrillation (AF),^1–3^ with pulmonary vein (PV) isolation (PVI) being the mainstay strategy of any first-time ablation procedure. Pulmonary vein isolation is commonly achieved using thermal energy sources [e.g. radiofrequency (RF), cryothermy and laser] either via point-by-point ablation or single-shot balloon devices. Single-shot technologies have been developed with the aim of simplifying AF ablation and improve its reproducibility; the most common energy source for balloon-based ablation is cryogenic, which has been proven to be non-inferior to RF ablation with respect to safety and efficacy of PVI.^4^ However, all above-mentioned thermal energy sources’ drawback is their lack of tissue selectivity, as they may elicit varying degrees of collateral damage to adjacent tissues and a non-negligible risk of serious complications [e.g. phrenic nerve (PN) palsy and atrio-oesophageal fistula].^5,6^

Pulsed field ablation (PFA) is an innovative technology recently adopted for the treatment of cardiac arrhythmias, which relies on the application of high-voltage, rapidly alternating electric fields to the heart tissue; pulse delivery results in nanopore development on cardiac cell membrane ultimately leading to apoptosis.^7^ Unlike other energy sources, PFA-induced lesion formation occurs in a non-thermal and selective fashion; specifically, waveforms can be tailored to preserve the surrounding tissues and concomitantly improve lesion durability.^8^ Preferential myocardial ablation by PFA has been extensively proven in preclinical and clinical studies demonstrating lower vulnerability of nerves, vasculature and oesophageal tissue to PFA. Several single-shot and focal ablation devices with PFA capabilities have been developed in the last years.^9–11^ Among them, the single-shot pentaspline has been the first PFA technology to receive regulatory approval (CE-mark). The system efficacy and safety have been described in several clinical studies as well as in a randomized trial.^12–14^ Nevertheless, to date, no study proved better safety and efficacy of PFA compared with other systems. In this perspective, we performed a systematic review and meta-analysis to compare the efficacy, safety, and procedural data of the PFA single-shot pentaspline system with available cryoballoon ablation (CRYO) technologies.

## Methods

### Data sources and searches

We systematically searched Medline, Cochrane, Journals@Ovid, and Scopus electronic databases for studies published from inception to 15 January 2024 and focusing on AF ablation employing the pentaspline Farapulse™ (Boston Scientific Inc., Marlborough, MA, USA) PFA catheter or second-/third-/fourth-generation CRYO (Medtronic: Arctic Front Advance™, Arctic Front Advance ST™, and Arctic Front Advance Pro™; Boston Scientific: POLARx™ and POLARx FIT™). The Farapulse™ system consists of a 12 F over-the-wire ablation catheter (Farawave™) that is advanced into the left atrium (LA) through a 13.8 F steerable sheath (Faradrive™) and a generator (Farastar™) that creates high-voltage electric fields (1.8–2.0 kV) (see Supplementary material online, *Figure S1*).

Three investigators (D.G.D.R., G.V., and A.P.) independently performed searches including the following terms: atrial fibrillation, pulsed field ablation, cryoballoon, catheter ablation, and pulmonary vein isolation. Detailed information on our literature search strategy is available in the Expanded Methods in Supplementary material.

### Study selection and outcomes

The Preferred Reporting Items for Systematic Reviews and Meta-Analyses statement for reporting systematic reviews and meta-analyses was used in this study.^15^ The predefined protocol was registered into the international prospective registry of systematic reviews PROSPERO (ID: CRD42023460640).

The studies had to fulfil the following criteria to be included in the analysis: (i) use of pentaspline Farapulse™ PFA catheter or second-/third-/fourth-generation CRYO for AF ablation, (ii) adult (>18 years old) study population, and (iii) description of at least one clinical outcome of interest. In case of multiple publications from the same centre, the study period was assessed. In studies with overlapping populations, the study with the largest sample size was included.

Editorials, surveys, case reports, reviews, expert opinions, and non-English studies were excluded.

### Data extraction and quality appraisal

Three investigators (D.G.D.R., G.V., and A.P.) extracted data from each study using standardized protocol and reporting forms. Three reviewers (D.G.D.R., G.V., and A.P.) independently assessed the quality items, and disagreements were resolved by consensus.

Individual patient data (IPDs) were retrieved from Kaplan–Meier plots if available. Data were extracted using a two-stage approach as described by Liu *et al*.^16^ In the first step, the Kaplan–Meier curves were digitized with a dedicated software (WebPlotDigitizer, https://apps.automeris.io/wpd/), where the axes were defined and the raw data coordinates [time and probability of freedom from atrial tachyarrhythmias (ATs)] were extracted in each of the Kaplan–Meier curves. In the second phase, the data coordinates were processed on the basis of the raw data coordinates from the first phase in combination with the numbers at risk at certain time points and/or the total number of patients and IPDs were reconstructed. Finally, the extracted IPDs from all studies were merged to create the study data set.

The quality of individual studies was assessed by three investigators (D.G.D.R., G.V., and A.P.) using the Newcastle–Ottawa Quality Assessment Scale for Cohort Studies and the Cochrane risk-of-bias tool for randomized clinical trials (RCTs).

### Study endpoints

The primary endpoints were acute procedural success assessed on a PV basis, as well as per patient. Procedural success on a PV basis was defined as the number of PVs successfully isolated with the chosen technology, without the need for touch-up applications with another device, divided by the overall number of PVs targeted for isolation. Procedural success per patient was defined as the number of patients with complete successful PVI with a certain technology, without the need for touch-up applications with another device, divided by the number of patients undergoing PVI. Safety endpoints were defined as follows: (i) overall periprocedural complications, which included ST-elevation, oesophageal lesions, stroke and/or transient ischaemic attack (TIA), pericardial tamponade or effusion with or without need for pericardiocentesis, transient or persistent PN palsy, death, and bleeding/vascular complications and (ii) major periprocedural complications, which included stroke and/or TIA, pericardial tamponade or effusion with the need for pericardiocentesis, persistent PN palsy, and death.

Secondary outcomes were procedural time, fluoroscopy time for PVI-only procedures, and freedom from AT recurrences at 12-month follow-up based on the reconstructed IPD in paroxysmal AF patients with only PVI ± cavotricuspid isthmus (CTI) ablation. Recurrence was defined as any documented AT episodes lasting >30 s after a blanking period of 90 days.

### Statistical analysis

Descriptive statistics are presented as means and standard deviations for continuous variables or a number of cases (*n*) and percentages (%) for dichotomous and categorical variables.

We used the restricted maximum likelihood method with the random-effects model to combine the untransformed raw proportions. The heterogeneity across studies was evaluated by using the χ^2^, *τ*^2^, and Higgins-*I*^2^ statistics. Random-effects models weighted by inverse variance were used because of clinical heterogeneity. The subgroup analyses are reported in Expanded Methods in Supplementary material. Individual patient data were represented through Kaplan–Meier curves and groups were compared with log-rank test. The hazard ratio (HR) with 95% confidence interval (CI) for the difference between the two groups was calculated using the Cox regression. Meta-regression analyses were performed to evaluate the sources of heterogeneity of efficacy endpoint (acute procedural success assessed per patient) and overall complications using Meta Regress command of STATA. Publication bias was assessed by graphical inspection of funnel plots. Statistical significance was defined as a two-tailed *P*-value < 0.05. Statistical analysis was performed using the meta function of STATA version 18.

## Results

### Study selection

Among 322 screened articles for PFA, 50 full texts were retrieved and reviewed for possible inclusion. A total of 20 studies fulfilled the selection criteria and were included in the final analysis (see Supplementary material online, *Figure S2A*). Among 1990 screened articles for CRYO, 488 full texts were retrieved and reviewed for possible inclusion; a total of 70 studies fulfilled the selection criteria and were included in the final analysis (see Supplementary material online, *Figure S2B*).

### Baseline characteristics

The 20 PFA studies enrolled 2481 patients, whereas the 69 CRYO studies included 13 766 patients.

Pulsed field ablation patients were 65.9% (95% CI: 58.3–74.1) males with an average age of 65.3 years (95% CI: 62.7–68.0); 58% (95% CI: 35–81) of them were affected by paroxysmal AF. Cryoballoon ablation patients were 65.8% (95% CI: 63.7–67.9) males with an average age of 62.4 years (95% CI: 60.3–64.5); 76% (95% CI: 70–82) of them had a diagnosis of paroxysmal AF. Pulsed field ablation patients had a mean CHA_2_DS_2_-VASc score of 2.2 (95% CI: 1.91–2.49), left ventricular ejection fraction (LVEF) of 57.3% (95% CI: 55.9–58.7), and a LA diameter of 41.8 mm (95% CI: 40.8–42.9). Cryoballoon ablation patients showed a CHA_2_DS_2_-VASc score of 1.81 (95% CI: 1.62–2.01), LVEF of 59.8% (95% CI: 58.9–60.8), and LA diameter of 39.7 mm (95% CI: 38.9–40.6).

Further details on baseline characteristics are reported in Supplementary material online, *Tables S1* and *S2*.

### Primary endpoints

**Figure 1:**
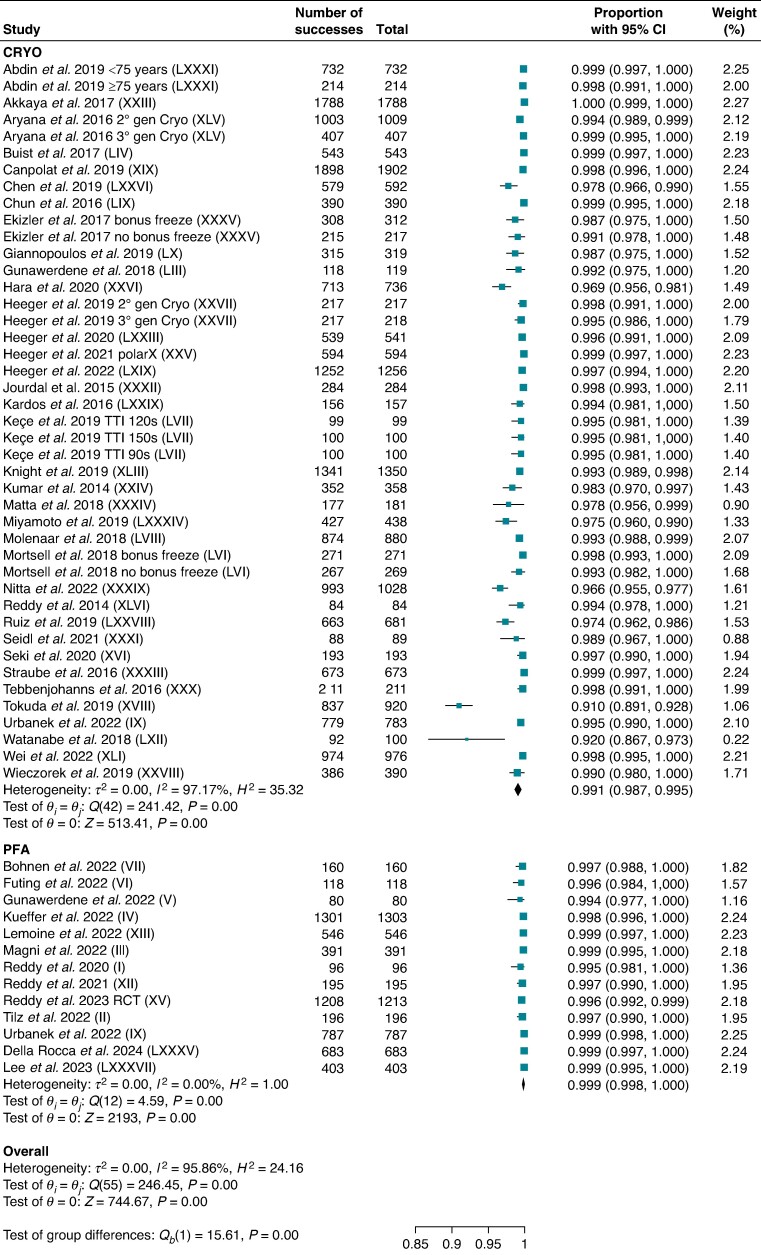
Acute procedural success per vein. Forest plot comparing acute procedural success per vein between PFA and CRYO. References in brackets are reported in Supplementary material. CI, confidence interval; CRYO, cryoballoon ablation; PFA, pulsed field ablation.

Thirteen studies for PFA and 35 others for CRYO reported acute procedural success per vein including 6171 and 21 952 veins, respectively. The PVI confirmation method of PFA studies is reported in Supplementary material online, *Table S3*. Pulsed field ablation demonstrated greater acute PVI success on a PV basis compared with CRYO [99.9% (95% CI: 99.8–100) vs. 99.1% (95% CI: 98.7–99.5); *P* < 0.001; *I*^2^ = 95.9%] (*Figure 1*).

**Figure 2:**
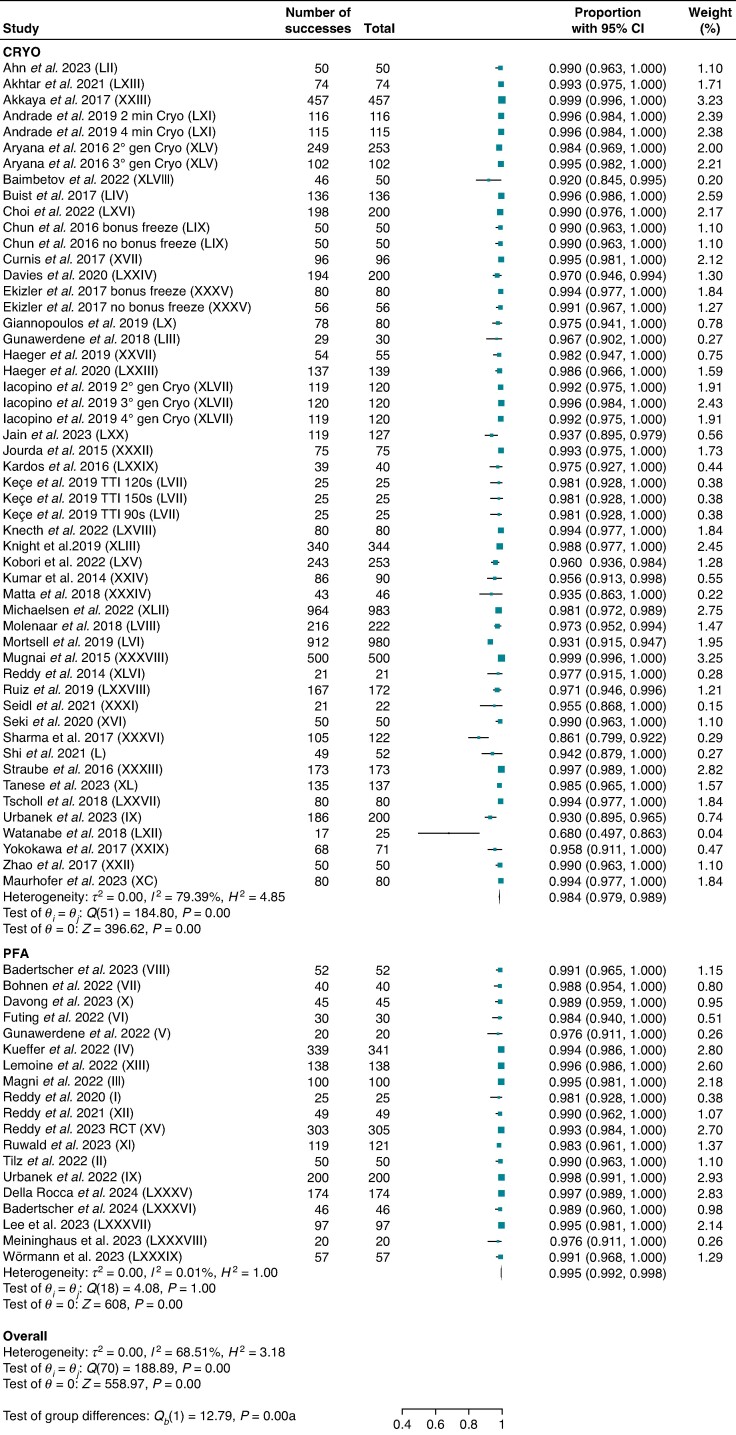
Acute procedural success per patient. Forest plot comparing acute procedural success per patient between PFA and CRYO. References in brackets are reported in Supplementary material. CI, confidence interval; CRYO, cryoballoon ablation; PFA, pulsed field ablation.

Nineteen studies for PFA and 43 others for CRYO reported acute procedural success on a patient basis including 1910 and 7819 patients, respectively. Pulsed field ablation yielded higher procedural success compared with CRYO [99.5% (95% CI: 99.2–99.8) vs. 98.4% (95% CI: 97.9–98.9); *P* < 0.001; *I*^2^ = 68.5%] (*Figure 2*). Subgroup analysis including only studies with PVI without additional lesions in the LA or with entrance and exit block PVI confirmation method confirmed greater procedural success for PFA compared with CRYO on both a patient and PV basis (see Supplementary material online, *Figures S3* and *S4*). Moreover, subgroup analysis including only studies with fourth-generation cryoballoons confirmed greater procedural success for PFA compared with CRYO on a patient basis (see Supplementary material online, *Figure S5*). No reduction in heterogeneity was found in subgroup analyses including only studies with >100 patients or RCTs (see Supplementary material online, *Figures S6* and *S7*).

Meta-regression analysis results were reported in Expanded Results section in Supplementary material.

### Safety endpoints

**Figure 3:**
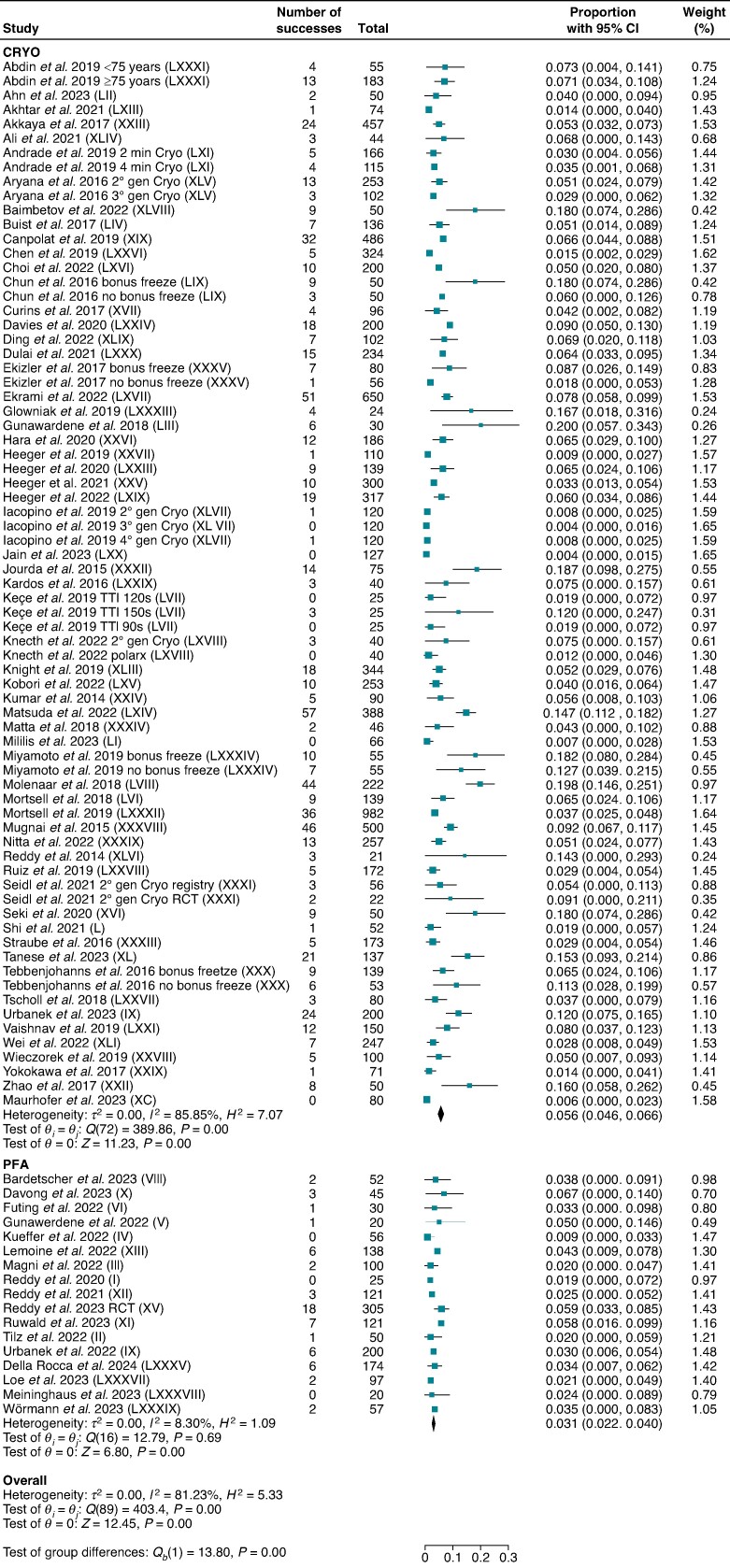
Overall periprocedural complications. Forest plot comparing overall periprocedural complications between PFA and CRYO. References in brackets are reported in Supplementary material.

Sixteen studies for PFA and 60 others for CRYO reported overall periprocedural complications in 1611 and 11 326 patients, respectively. Pulsed field ablation demonstrated lower overall periprocedural complications compared with CRYO [3.1% (95% CI: 2.2–4.0) vs. 5.6% (95% CI: 4.6–6.6); *P* < 0.001; *I*^2^ = 81.2%] (*Figure 3*).

The pooled incidence of periprocedural complications in each group is shown in Supplementary material online, *Table S4*. Among PFA patients, the most common complications were bleeding/vascular complication [1.30% (95% CI: 0.60–1.90)], followed by ST-elevation due to coronary spasm or air embolism [0.85% (95% CI: 0.00–2.20)]. Pericardial tamponade or effusion with the need for pericardiocentesis was reported in 0.80% (95% CI: 0.28–1.33) of patients. In addition, stroke and/or TIA occurred in 0.39% (95% CI: 0.00–1.11) while PN palsy in 0.01% (95% CI: 0.00–0.07) of patients.

Among CRYO patients, the most common complications were PN palsy [1.84% (95% CI: 1.58–2.10)] and PN capture monitoring method during right PV ablation is reported in Supplementary material online, *Table S5*. Bleeding/vascular complication and stroke and/or TIA were reported in 1.20% (95% CI: 0.90–1.50) and 0.45% (95% CI: 0.29–0.62) of patients, respectively. In addition, pericardial tamponade or effusion with the need for pericardiocentesis occurred in 0.40% (95% CI: 0.24–0.56) while ST-elevation due to coronary spasm or air embolism in 0.33% (95% CI: 0.03–0.62) of patients. Oesophageal lesions were reported in five studies of the CRYO group, but none of these studies reported an atrio-oesophageal fistula event.

Meta-regression analysis results were reported in Expanded Results section in Supplementary material. Subgroup analysis was performed in the CRYO group to assess the influence of additional/bonus complications on overall complications outcome showing a non-statistically significant difference (Supplementary material online, *Figure S8*). Subgroup analysis including only studies with fourth-generation cryoballoons confirmed lower overall periprocedural complications for PFA compared with CRYO (see Supplementary material online, *Figure S9*).

**Figure 4:**
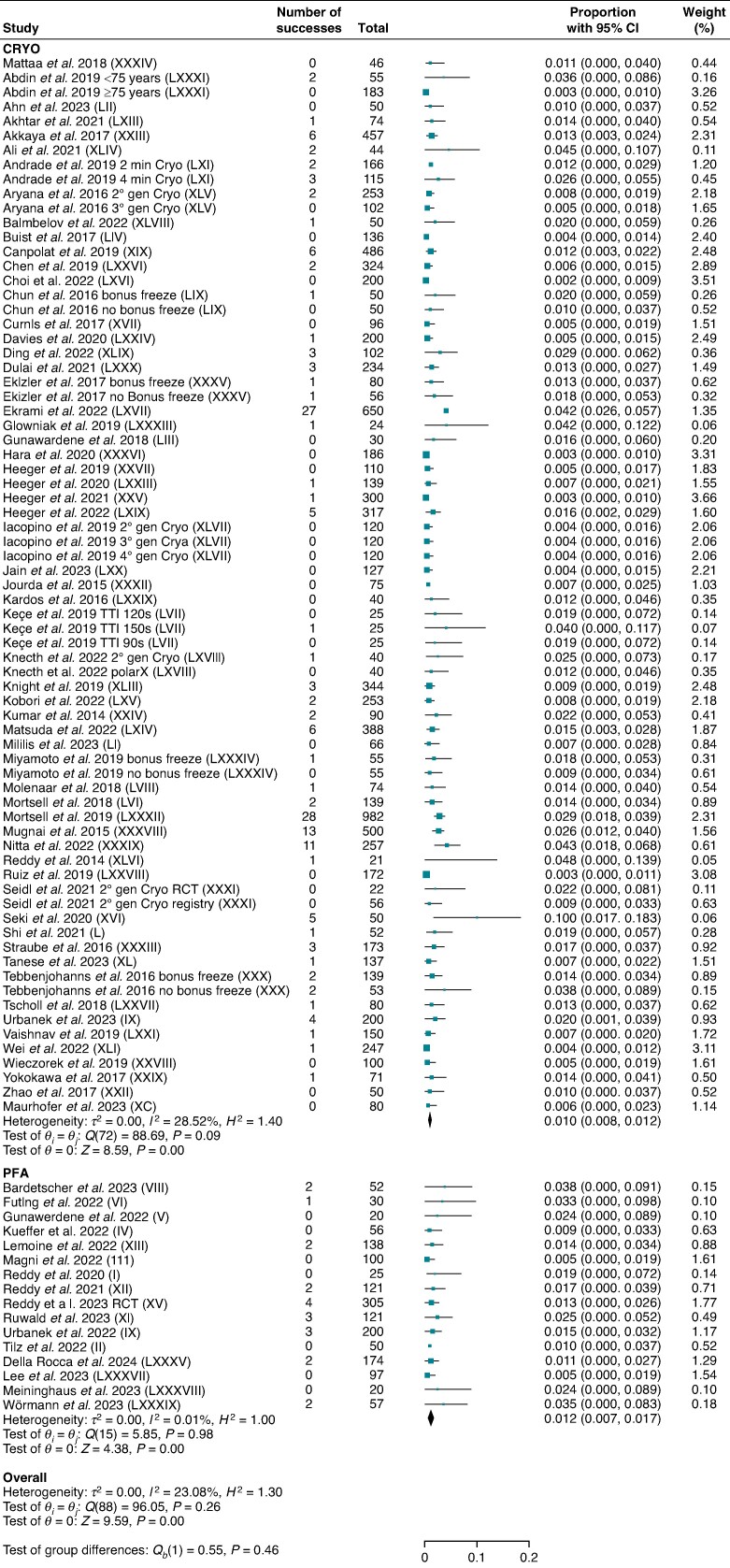
Major periprocedural complications. Forest plot comparing major periprocedural complications between PFA and CRYO. References in brackets are reported in Supplementary material. CI, confidence interval; CRYO, cryoballoon ablation; PFA, pulsed field ablation.

Sixteen studies for PFA and 40 studies for CRYO reported major periprocedural complications including 1566 and 11 178 patients, respectively. No difference was found between the two ablation strategies [1.2% (95% CI: 0.7–1.7) vs. 1.0% (95% CI: 0.8–1.2); *P* = 0.46; *I*^2^ = 23.1%] (*Figure 4*). No significant reduction in heterogeneity was found in any subgroup analyses (see Supplementary material online, *Figures S6*, *S7*, and *S10*).

### Secondary endpoints

**Figure 5:**
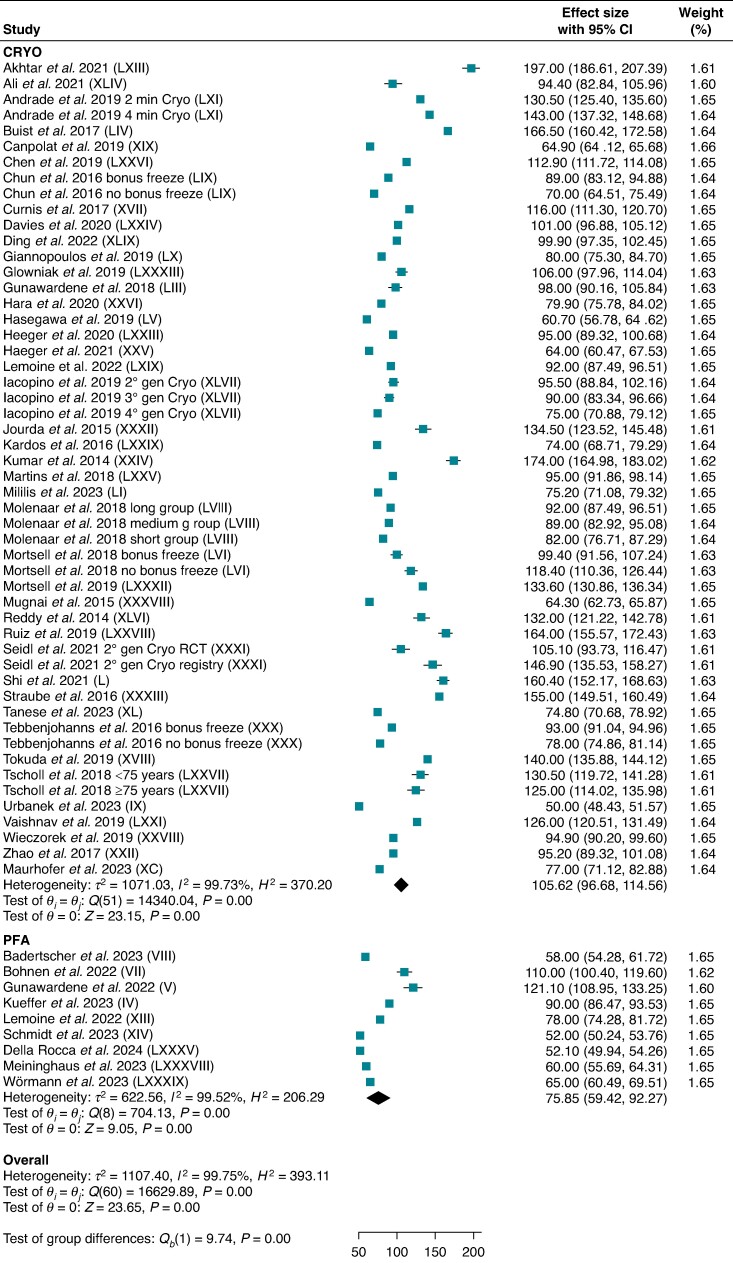
Procedural time. Forest plot comparing procedural time between PFA and CRYO. References in brackets are reported in Supplementary material. CI, confidence interval; CRYO, cryoballoon ablation; PFA, pulsed field ablation.

Nine studies for PFA and 42 studies for CRYO reported procedural time for PVI-only procedures, including 1371 and 5641 patients, respectively. Pulsed field ablation demonstrated shorter procedural time compared with CRYO [75.9 min (95% CI: 59.4–92.3) vs. 105.6 min (95% CI: 96.7–114.6); *P* < 0.001; *I*^2^ = 99.8%] (*Figure 5*).

**Figure 6:**
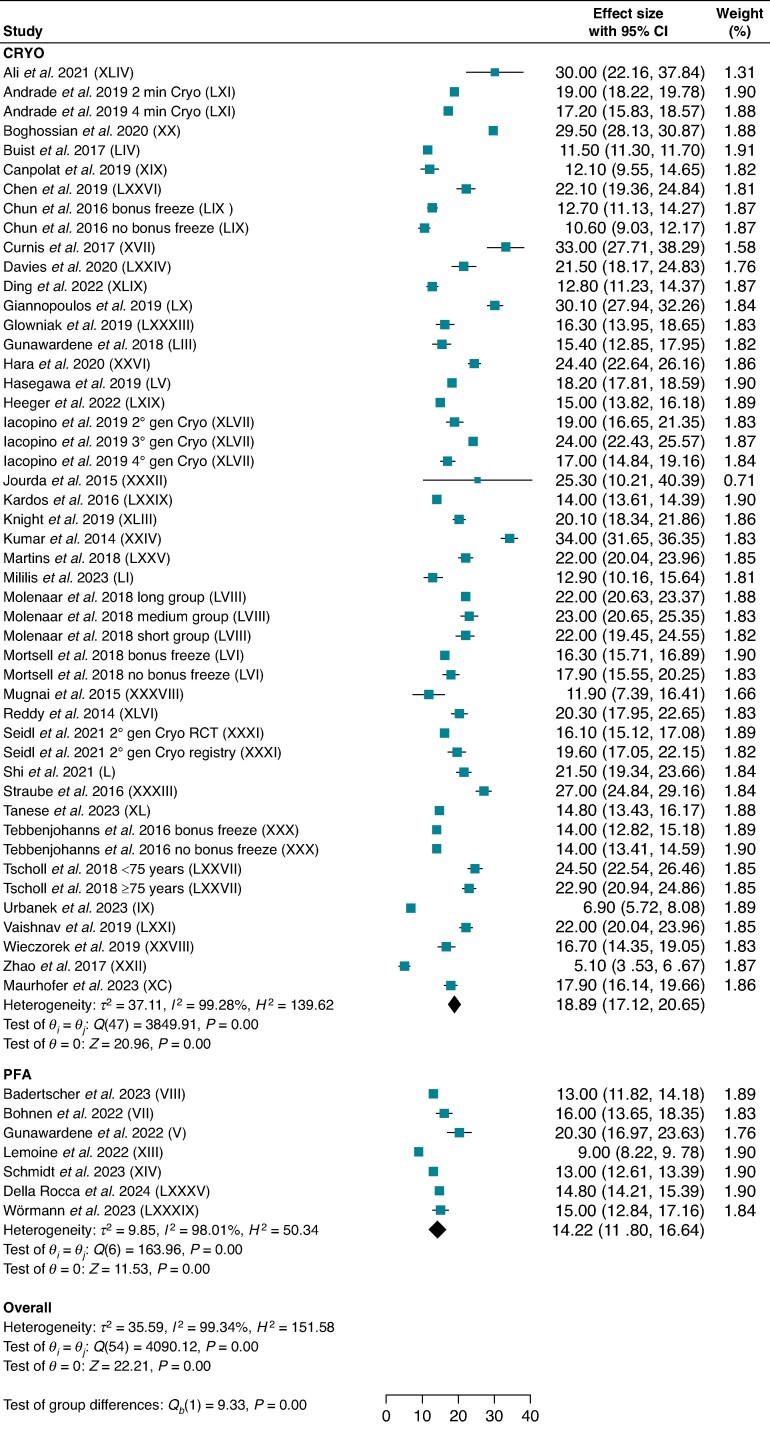
Fluoroscopy time. Forest plot comparing fluoroscopy time between PFA and CRYO. References in brackets are reported in Supplementary material. CI, confidence interval; CRYO, cryoballoon ablation; PFA, pulsed field ablation.

Seven studies for PFA and 37 studies for CRYO reported fluoroscopy times for PVI-only procedures, including 1314 and 5781 patients, respectively. Pulsed field ablation demonstrated shorter fluoroscopy time compared with CRYO [14.2 min (95% CI: 11.8–16.6) vs. 18.9 min (95% CI: 17.1–20.7); *P* < 0.001; *I*^2^ = 99.3%] (*Figure 6*). Subgroup analysis including only studies with fourth-generation cryoballoons confirmed lower fluoroscopy time for PFA compared with CRYO (see Supplementary material online, *Figure 1^1^*).

### Individual patient data analysis

**Figure 7:**
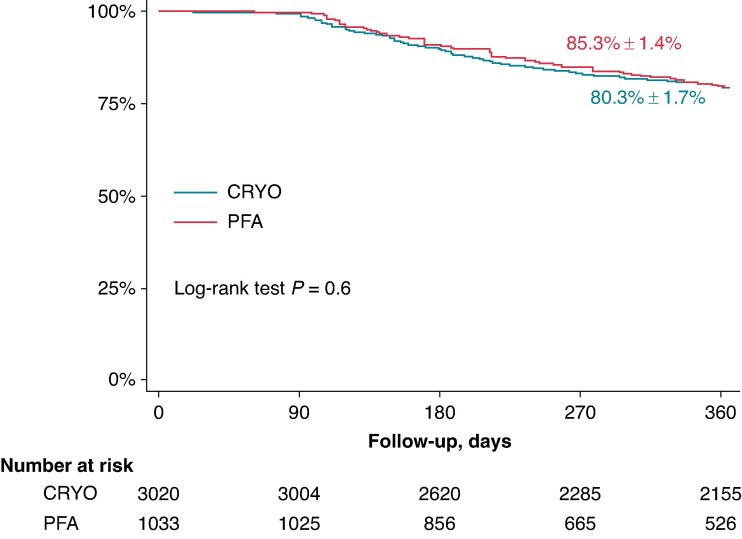
Kaplan–Meier survival estimates for atrial tachycardia recurrences comparing PFA and CRYO. CRYO, cryoballoon ablation; PFA, pulsed field ablation.

Five studies for PFA and 21 studies for CRYO presented a Kaplan–Meier curve depicting AT-free survival in paroxysmal AF patients with PVI (±CTI) only procedures. The IPDs of 4076 patients were constructed, 1033 in the PFA group and 3043 in the CRYO one. The follow-up of the studies included in the IPD analysis is reported in Supplementary material online, *Table S6*. Atrial tachyarrhythmia freedom at 1-year follow-up was 82.3% ± 1.4% vs. 80.3% ± 1.7%, respectively (log-rank *P* = 0.61) (*Figure 7*). Neither CRYO nor PFA showed any additional advantages for AT recurrence at Cox regression analysis [HR: 0.96 (95% CI: 0.81–1.13), *P* = 0.61].

### Publication bias

A graph and summary of the Newcastle–Ottawa Quality Assessment Scale for Cohort Studies for each observational study and Cochrane risk-of-bias tool for RCTs for PFA and CRYO are reported in Supplementary material online, *Figures 12–15*. The funnel plots for visual inspection of the bias are reported and showed no significant bias in Supplementary material online, *Figure 1^6^*.

## Discussion

Herein, we report the first meta-analysis performing a comparison of the efficacy, safety, and acute procedural outcomes of PFA vs. CRYO in AF ablation (*Graphical Abstract*).

The main findings are as follows:

1. Pulsed field ablation via the pentaspline catheter contributed to greater acute procedural success, assessed either on a PV and a patient basis, compared with CRYO.
2. Pulsed field ablation-based procedures showed a shorter procedural time and X-ray exposure.
3. Pulsed electric field (PEF) demonstrated a remarkable safety profile, as demonstrated by a significantly lower incidence of overall complications, with PN palsy, either transient or persistent, being the main determinant of this difference; no difference was found for major periprocedural complications.
4. Freedom from ATs at 1 year was high (>80.0%) and similar between technologies.

Single-shot, cryogenic-based devices have been developed with the aim of achieving PVI in a simple and reproducible manner, thereby limiting the need for extensive training and the inter-operator variability. Previous studies have confirmed a high safety profile of cryoballoon, as well as non-inferiority compared with focal RF ablation.^4^

Pulsed field ablation is a novel form of non-thermal energy for AF ablation, which relies on rapidly alternating high electric fields and ultimately cell membrane nanopore formation, namely electroporation, to achieve cell death and lesion formation.^8,17–19^ Unlike cryogenic energy, PEF can be tailored to improve lesion durability and tissue selectivity, thereby targeting cardiomyocytes and sparing other tissues (e.g. nerves, oesophagus, and blood vessels). The effect on cardiac tissue appears to be long-lasting, as confirmed by remapping data showing a high percentage of long-lasting PVI.^8^ The Farapulse™ system is the first PFA device to have received regulatory approval, as well as the only one with ‘single-shot’ features.

Ours is the first meta-analysis to compare procedural success, safety, and long-term freedom from arrhythmia between cryothermy- vs. PEF-based single-shot devices. Our findings confirm a high success rate of acute PVI for both techniques; however, this is the first study to demonstrate greater acute procedural success with PFA compared with CRYO. Acute procedural success was assessed either on a PV basis as well as per patient and referred to a successful PV isolation without the need to adopt a different ablation technology. Even if the difference in acute procedural success on PV basis was only ≈1%, this may lead to a clinically meaningful issue requiring a switch to another ablation system with increased procedural costs and time. The superior acute efficacy of PFA may be explained by a greater adaptability of the Farapulse™ system to the variable PV anatomy compared with the CRYO, as a result of the dual configuration of basket and flower.^20^ The flower configuration allows for a wide antral circumferential area of the lesion, as evidenced by ultra-high-density mapping studies showing greater coverage even in PVs with complex anatomy.^21^ For these reasons, studies have shown a smooth transition from cryoballoon to PFA and a steep learning curve for operators who are new to single-shot technologies.^20,22^

Achieving isolation of the right PVs with cryoballoon may be hindered by the vicinity of the PN, with PN palsy, either transient or persistent, being the most common periprocedural complication in such procedures.^23–25^ Periprocedural PN palsy is a potential cause of incomplete right PV isolation requiring point-by-point ablation. In patients treated with PFA, the incidence of transient PN palsy is extremely rare (0.3%–0.46%), whereas PFA-related persistent PN injury has been reported only once in the literature.^13,26^

Our findings also underlined the remarkable safety profile of PFA, as suggested by a significantly lower rate of overall periprocedural complications compared with CRYO (3.1% vs. 5.6%); this difference was mainly due to the rate of PN injury in the CRYO group (1.84% vs. 0.01%). Although the rate of major periprocedural complications was similar, it is important to highlight that PEF does not pose any risks of thermal lesions to the oesophagus, thereby completely eliminating the risk of the dreaded and life-threatening atrio-oesophageal fistula.^6^

Our meta-analysis also demonstrated shorter procedural (75.9 min vs. 105.6 min) and fluoroscopy (14.2 min vs. 18.9 min) times with PFA. A reduction in procedure times was also demonstrated in the ADVENT with PFA compared with conventional thermal energy, but this was mainly due to the use of electroanatomical mapping and longer ablation times in the point-by-point RF ablation subgroup.^14^ In contrast with our findings, the ADVENT trial reported a longer fluoroscopy time with PFA, but, again, this was probably related to the ubiquitous use of non-fluoroscopic electroanatomical mapping systems with RF ablation.^14^ As a matter of fact, CRYO showed significantly higher X-ray exposure when compared with RF in the FIRE AND ICE randomized trial.^4^ Operator experience was proven to decrease fluoroscopy time with PFA; therefore, the integration of the Farapulse™ system with 3D mapping will soon provide an alternative for catheter localization during PFA.^27^

### Limitations

Our meta-analysis has several limitations. (i) Observational studies were included in this analysis, which introduced all the inherent limitations and biases related to their design. In particular, studies of cryoablation and PFA were non-contemporaneous, and each study examined only one ablation modality or the other. None of the studies included in this meta-analysis provided a head-to-head comparison of the two modalities. (ii) High levels of heterogeneity for efficacy and safety outcomes were observed. (iii) Data on acute procedural success per vein were available only in a limited number of studies, and the strategy to confirm PV isolation was not uniform among them. (iv) A large registry such as MANIFEST-PF^28^ was excluded from this study due to overlap with data from the EU-PORIA registry. (v) The studies available for PFA report data from a first experience with PFA and not from operators with long experience, as for CRYO. This factor may have impacted procedural duration, as well as major complication rates. Similarly, the CRYO group includes studies published since 2014; improved materials and technologies might have contributed to shorter procedural and dwelling times in recent years. (vi) Finally, in our meta-analysis, we showed a higher number of persistent AF in the PFA group than in the CRYO group (76% vs. 58%). The reason for the higher number of persistent AF patients in the PFA group is due to the capability of the Farapulse™ catheter with the flower shape to perform not only PVI but also posterior wall isolation and posterior mitral line ablation.

## Conclusions

In our meta-analysis, PFA contributed to greater acute procedural success and a better safety profile than CRYO. In addition, PFA led to shorter procedural and fluoroscopy times.

## Supplementary Material

euae293_Supplementary_Data

## Data Availability

The data underlying this article will be shared on reasonable request to the corresponding author.

## Appendix

HRMC Investigators: Alvise Del Monte, MD; Sahar Mouram, MD; Cinzia Monaco, MD; Ilenia Lombardo, MD; Roberto Scacciavillani, MD; Kazutaka Nakasone, MD; Maria Cespon Fernandez, MD, PhD.
