# Supplementary material for: Multielectrode catheter-based pulsed electric field vs. cryoballoon for atrial fibrillation ablation: a systematic review and meta-analysis
Source: Europace. 2024 Nov 23;26(12):euae293. doi: 10.1093/europace/euae293 (PMC11641428; doi:10.1093/europace/euae293)
Supplement: euae293_Supplementary_Data [file euae293_supplementary_data.docx]

**SUPPLEMENTAL MATERIALS**

**Multielectrode Catheter-Based Pulsed Electric Field Versus Cryoballoon for Atrial Fibrillation Ablation: a Systematic Review and Meta-Analysis**

**Expanded Methods**

Data Sources and Searches

**Expanded Results**

**Supplemental Tables**

Supplemental Table 1

Supplemental Table 2

Supplemental Table 3

Supplemental Table 4

Supplemental Table 5

Supplemental Table 6

Supplemental Table 7

**Supplemental Figures**

Supplemental Figure 1

Supplemental Figure 2

Supplemental Figure 3

Supplemental Figure 5

Supplemental Figure 6

Supplemental Figure 7

Supplemental Figure 8

Supplemental Figure 9

Supplemental Figure 10

Supplemental Figure 11

Supplemental Figure 12

**Expanded Methods**

*Data Sources and Searches*

We used the following search strings for PFA respectively for Pubmed (1), Embase (2), Scopus (3):

1) (((((Atrial Fibrillation) AND (Pulsed Field Ablation)) OR (PFA)) OR (Non thermal)) OR (Atrial Fibrillation Ablation))))

2) (((Mesh descriptor: [Pulsed Field Ablation] explode all trees) OR ((Catheter ablation) :ti OR (PFA and Atrial Fibrillation n):ti OR (Non thermal) OR (Atrial Fibrillation Ablation)))

3) (Pulsed Field Ablation or PFA).ti. and (PFA and Pulmonary Vein Isolation or PVI or Atrial Fibrillation Ablation).ab.

We used the following search strings for CRYO respectively for Pubmed (1), Embase (2), Scopus (3):

1) (((((Atrial Fibrillation) AND (Cryoballoon Ablation)) OR (Cryoballoon)) OR (Cryo)) OR (Atrial Fibrillation Ablation))))

2) (((Mesh descriptor: [Cryoballoon Ablation] explode all trees) OR ((Catheter ablation) :ti OR (Cryoballoon and Atrial Fibrillation n):ti OR (Cryo) OR (Atrial Fibrillation Ablation)))

3) (Cryoballoon Ablation or Cryo).ti. and (Cryo and Pulmonary Vein Isolation or PVI or Atrial Fibrillation Ablation).ab.

*Statistical Analysis*

We performed subgroup analyses to assess potential sources of heterogeneity of primary and safety endpoints by including only studies with >100 patients or RCTs. Subgroup analysis was performed including only studies with 4^th^ generation cryoballoons. Further subgroup analysis was performed for Efficacy endpoints including only patients without additional lesions in the left atrium and with only Entrance and Exit block PVI confirmation method. Moreover, subgroup analysis was performed in the CRYO group to assess the influence of Additional/Bonus complications on Overall complications.

**Expanded Results**

At metaregression analysis for Efficacy endpoint no characteristic demonstrated influence (Supplemental Table 7).

At metaregression analysis for Overall complications only PFA energy demonstrated influence (β = -0.022 [-0.04– -0.002]; *I^2^*= 81.00%;p = 0.03) (Supplemental Table 8).

**Supplemental Table 1. Study Baseline Characteristics of Patients Included in the Analysis for Pulsed Field Ablation.**

| **First Author** | **Study design** | **Patients, n** | **PV, n** | **Power** | **Applications for PVI** | **Ablation Outside PVs** | **Age, mean ± SD (min–max)** | **PAF n, (%)** | **PerAF n, (%)** | **CHA2DS2‐VASc, mean ± SD (min–max)** | **LVEF %, mean ± SD (min–max)** | **LA diameter mm, mean ± SD (min–max)** | **Follow up,mean ± SD (min–max)** |
| --- | --- | --- | --- | --- | --- | --- | --- | --- | --- | --- | --- | --- | --- |
| **Badertscher et al. 2023 [VIII]** | PS-SC | 52 | NA | NA | NA | NA | NA | NA | NA | NA | NA | NA | 6 mo |
| **Bohnen et al. 2022 [VII]** | PS-SC | 40 | 160 | 2.0 kV for PVI | 4x4 per vein | NA | 62 ± 9 | 25 (62) | 15 (38) | NA | 58 ± 4 | 41 ± 4 | 190 (167-202) d |
| **Davong et al. 2023 [X]** | PS-SC | 45 | NA | NA | 4x4 per vein | PWI+PML 45 (100) | 67.1±10 | 0 | 45(100) | NA | 56.5 ± 11.2 | NA | 107.8 ± 59.5 d |
| **Füting et al. 2022 [VI]** | PS-SC | 30 | 118 | 1.9 kV for PVI | 4x4 per vein | NA | 63 ± 10 | 30 (100) | 0 | 2 (1–3) | 60 ± 6 | 43 ± 6 | 90 d |
| **Gunawardene et al. 2022 (PVI) [V]** | PS-SC | 11 | 44 | 1.9 kV for PVI | 4x4 per vein | NA | 75.2 ± 6.2 | 7 (63) | 4 (37) | 3 (2–3.5) | 10 (90.9): 50-70 0: 40-49 1 (9.1): 30-39 0: <30 | 45.2 ± 4.1 | NA |
| **Gunawardene et al. 2022 (PVI+PWI) [V]** | PS-SC | 9 | 36 | 1.9 kV for PVI | 4x4 per vein | PWI 9 (100) | 64.4 ± 10.2 | 0 | 9 (100) | 2 (1–4) | 5 (55.6): 50-70 1 (11.1): 40-49 1 (11.1): 30-39 2 (22.2): <30 | 42.1 ± 5.8 | NA |
| **Kueffer et al. 2023 [IV]** | PS-SC | 341 | 1304 | 2.0 kV | 4x4 per vein | NA | 68 (60-74) | 156 (46) | 185 (54) | NA | 60 (50-60) | 43 (38-48) | 9 (6–14) mo |
| **Lemoine et al. 2022 [XIII]** | PS-DC | 138 | 546 | 1.8-2.0 kV for PVI | 4x4 per vein | NA | 67 ± 12 | 52 (38) | 86 (62) | 2.7 ± 1.7 | 52 ± 10 | 43 ± 5 | 249 ± 90 d |
| **Magni et al. 2022 [III]** | PS-SC | 100 | 391 | NA | 4x4 per vein | PWI 12 (12) | 62.9 ± 9.4 | 80 (80) | 20 (20) | 1.5 (1.0–2.0) | 54.7 ± 3.7 | NA | 90.5 (83–98) d |
| **Reddy et al. 2020 [I]** | PS-SC | 25 | 96 | 1.6-2.0 kV | 4x4 per vein | PW 24(100)  CTI 13(54) | 67 (60–70) | 0 | 25 (100) | NA | 60 (51–65) | 4.3 (4.2–4.7) | 82 (76-90) d |
| **Reddy et al. 2021 [XII]** | PS-MC | 49 | 195 | 1.8-2.0 kV | 4x4 per vein | CTI 4 (3.3) | 56.9 ± 10.4 | NA | NA | NA | 61.2 ± 7.2 | 40.0 ± 5.0 | 12 mo |
| **Reddy et al. 2023 [XV]** | RCT-MC | 305 | 1213 | 1.8-2.0 kV | 4x4 per vein | NA | 62.4±8.7 | 305 (100) | 0 | 1.7 ± 1.2 | NA | 38.8 ± 5.7 | 12mo |
| **Ruwald et al. 2023 [XI]** | PS-SC | 121 | NA | 2 kV | 4x4 per vein | NA | 63±11 | 71 (59) | 50 (41) | 1.8±1.4 | 55±9 | 39±7 | 308±87 d |
| **Schmidt et al. 2023 [XIV]** | Registry-MC | 1233 | 4872 | 1.8-2.0 kV for PVI | 4x4 per vein | PWI127 (10.3)  PML 62 (5)  CTI 6(0.5) | 66 ± 11 | 740 (60) | 493 (40) | 2.3 ± 1.6 | 57 ± 10 | NA | 365 (323-386) d |
| **Tilz et al. 2023 [II]** | PS-SC | 50 | 196 | 2.0 kV | 4x4 per vein (bonus) | NA | 63.6 ± 10.7 | 28 (56) | 22 (44) | 1.7 ± 1.4 | NA | 40 ± 7.4 | NA |
| **Urbanek et al. 2023 [IX]** | PS-SC | 200 | 787 | 2.0 kV for PVI | 4x4 per vein | NA | 71 (62–77) | 116 (58) | 84 (42) | 2 (2–4) | NA | 41 (37–46) | 379 (366–404) d |
| **Della Rocca et al. 2024 [LXXXV]** | PSM-MC | 174 | 683 | 2.0 kV for PVI | 4x4 per vein | NA | 62 ± 11.6 | 174 (100) | 0 (0) | 2 (1-3) | 59.4 ± 4.2 | 41.8 ± 4.9 | 12.3 ± 2.1 m |
| **Badertscher et al. 2024 [LXXXVI]** | RS-SC | 100 | NA | 2.0 kV for PVI | 4x4 per vein | PW 100(100) | 69 (63–75) | 45 (45) | 55(55) | NA | 55 (45–60) | 43 (39–47) | 139 (89–274) d |
| **Lee et al. 2023 [LXXXVII]** | PS-SC | 97 | 403 | 2.0 kV for PVI | 4x4 per vein | PW 25(25.8) | 62 (36–80) | 35 (36.1) | 62 (63.9) | 2 (0–5) | 57 (30–72) | NA | 218 (16–343) d |
| **Meininghaus et al. 2023 [LXXXVIII]** | PS-SC | 20 | NA | 2.0 kV for PVI | 4x4 per vein | NA | 71.5 (63.4/75.8) | 7 (35) | 13 (65) | NA | NA | 46.0 (42.5/49.5) | NA |
| **Wörmann et al. 2023 [LXXXIX]** | PS-SC | 57 | NA | 2.0 kV for PVI | 4x4 per vein | NA | 67 ± 13 | 17 (30) | 40 (70) | 3 | 56 ± 6 | 39.6 ± 6.0 | 125 (109–162) d |

***AF:*** *Atrial Fibrillation;* ***CTI:*** *Cavotricuspid Isthmus;* ***d:*** *day;* ***DC:*** *Double Center;* ***NA:*** *not available;* ***LVEF:*** *Left Ventricular Ejection Fraction;* ***mo:*** *months;* ***MC:*** *Multicenter;* ***PAF:*** *Paroxysmal Atrial Fibrillation;* ***PerAF****: Persistent Atrial Fibrillation;* ***PML:*** *Posterior Mitral Line* ***PS:*** *Prospective Study;* ***PV:*** *Pulmonary Vein;* ***PWI:*** *Posterior Wall Isolation;* ***RCT:*** *Randomized Clinical Trial;* ***SC:*** *Single Center;*

*References in brackets are reported in Supplementary Materials.*

**Supplemental Table 2. Study Baseline Characteristics of Patients Included in the Analysis for Cryoballoon Ablation**

| **First Author** | **Study design** | **Patients** | **Pulmonary veins** | **Device** | **Application duration** | **Age, mean ± SD (min–max)** | **PAF n, (%)** | **PerAF n, (%)** | **CHA2DS2‐VASc, mean ± SD (min–max)** | **LVEF %, mean ± SD (min–max)** | **LA diameter mm, mean ± SD (min–max)** | **Follow up,mean ± SD (min–max)** |
| --- | --- | --- | --- | --- | --- | --- | --- | --- | --- | --- | --- | --- |
| **Abdin et al. 2019 <75 years [LXXXI]** | RS-SC | 55 | 214 | 28 mm Arctic Front Advance | 1 application 180-240 s ± bonus applications | 78 ± 2.8 | 17 (31) | 38 (69) | 4.0 ± 1.3 | 51.6 ± 8.3 | 40.8.6 ± 5.5 | 12.4 ± 5.9 mo |
| **Abdin et al. 2019 ≥75 years [LXXXI]** | RS-SC | 183 | 732 | 28 mm Arctic Front Advance | 1 application 180-240 s ± bonus applications | 60.8 ± 9.5 | 74 (41) | 109 (59) | 2.0 ± 1.3 | 52.5 ± 8.0 | 40.8 ± 6.6 | 11.7 ± 5.3 mo |
| **Ahn et al. 2023 [LII]** | RCT-MC | 50 | NA | 28 mm Arctic Front Advance or Arctic Front Advance Pro | 1 application 180-240 s | 61.8 ± 9.8 | 50 (100) | 0 | 2.1 ± 1.7 | 62.5 ± 4.3 | 39.9 ± 6.9 | 680.9 ± 166.9 d |
| **Akhtar et al. 2021 [LXIII]** | RS-SC | 74 | NA | 28 mm Arctic Front Advance | 1 application 180 s ± bonus applications | 77.5 (76–80) | 53 (72) | 21 (28) | 4 (3–4) | 60 (55–65) | 4.31 ± 0.75 | 12 mo |
| **Akkaya et al. 2017 [XXIII]** | PS-SC | 457 | 1788 | 28 mm Arctic Front Advance | 1 application 180 s | 61 (54-68) | 278 (61) | 179 (39) | 1.5 ± 1.2 | 62 (57/62) | NA | 28 (15 ± 42) mo |
| **Ali et al. 2021 [XLIV]** | PS-MC | 44 | 174 | 28 mm Arctic Front Advance | 1 application 180-240 s | 53.8 ± 15.0 | 44 (100) | 0 | 1 (0–2) | 63.7 ± 5.3 | 37.6 ± 4.4 | 12 mo |
| **Andrade et al. 2019 2 min Cryo [LXI]** | RCT-MC | 115 | NA | 28 mm and 23 mm Arctic Front Advance | 1 application 120-240 s + bonus applications | 59.6 ± 9.9 | 109 (95%) | 6 (5) | 1 (0-2) | NA | 37.7±10.3 | 12 mo |
| **Andrade et al. 2019 4 min Cryo [LXI]** | RCT-MC | 116 | NA | 28 mm and 23 mm Arctic Front Advance | 1 application 120-240 s + bonus applications | 58.2 ± 10.7 | 113 (97) | 3 | 1 (0, 2) | NA | 38.2 ± 7.4 | 12 mo |
| **Aryana et al. 2016 2° gen Cryo [XLV]** | PS-MC | 253 | 1009 | 28 mm and 23 mm Arctic Front Advance | 1 application guided by TTI ± bonus applications | 64 ± 10 | 179 (71) | 74 (29) | NA | 55 ± 10 | 43 ±6 | 13 ± 2 |
| **Aryana et al. 2016 3° gen Cryo [XLV]** | PS-MC | 102 | 407 | 28 mm and 23 mm Arctic Front Advance | 1 application guided by TTI ± bonus applications | 64 ± 11 | 75 (74) | 28 (26) | NA | 56 ± 8 | 45 ± 6 | 10 ± 1 |
| **Baimbetov et al. 2022 [XLVIII]** | RCT-NA | 50 | 198 | 28 mm Arctic Front Advance | 1 application 240 s ± bonus applications | 61.3 ± 10.2 | 0 | 50 (100%) | 1 ± 0.5 | 59 ± 5 | 41 ± 5 | 24 mo |
| **Boghossian et al. 2020 [XX]** | PS-SC | 108 | NA | 28 mm Arctic Front Advance | 1 application 180 s ± bonus applications | 58 ± 13 | 65 (60) | 43 (39) | NA | NA | NA | 367 ± 20 d |
| **Buist et al. 2017 [LIV]** | RCT-SC | 136 | 543 | 28 mm Arctic Front Advance | 1 application 240 s ± bonus applications | 59.7 ± 9.9 | 117 (88) | 16 (12) | 1.37 ± 1.32 | NA | NA | 381 ( 292–563) d |
| **Canpolat et al. 2019 [XIX]** | PS-SC | 486 | 1902 | 28 mm Arctic Front Advance | 1 application 180-240 s ± bonus applications | 59.8 ± 10.9 | 344 (71) | 142 (29) | 1.93 ± 0.49 | 61.7 ± 6.9 | 38.5 ± 5.7 | 39 (26-56) |
| **Chen et al. 2019 [LXXVI]** | RS-SC | 324 | 592 | 28 mm Arctic Front Advance | At least two applications for 150-180 s. | 78.8 ± 2.4 | 216 (67) | 108 (33) | 3.09 ± 0.881 | 57.7 ± 5.9 | 42.1 ± 4.1 | 14.33 ± 9.07 mo |
| **Choi et al. 2022 [LXVI]** | RS-SC | 200 | NA | 28 mm Arctic Front Advance | 1 application 180 s ± bonus applications | 57.5 ± 9.8 | 120 (60) | 80 (40) | 1.3 ± 1.2 | 61.1 ± 7.1 | 42.3 ± 6.2 | 361 ± 180 d |
| **Chun et al. 2016 Bonus freeze [LIX]** | RCT-SC | 50 | 390 | 28 mm Arctic Front Advance | 1 application 240 s ± bonus applications | 66 ± 10 | 50 (100) | 0 | NA | NA | 41 ± 4 | 372 (351-455) d |
| **Chun et al. 2016 No Bonus freeze [LIX]** | RCT-SC | 50 | NA | 28 mm Arctic Front Advance | 1 application 240 s ± bonus applications | 63 ± 12 | 50 (100) | 0 | NA | NA | 39 ± 4 | 378 (361-483) d |
| **Curnis et al. 2017 [XVII]** | PS-SC | 96 | NA | 28 mm Arctic Front Advance | 1 application 180 s ± bonus applications | 57.4 ± 9.9 | 56 (58) | 40 (42) | 1 (0–2) | 57.9 ± 6.4 | 42.9 ± 5.8 | 416 (288–706) d |
| **Davies et al. 2020 [LXXIV]** | RS-SC | 200 | NA | 28 mm Arctic Front Advance | 1 application 240 s ± bonus applications | 61.8 (10.1) | 136 (68) | 64 (23) | NA | 61.3 ± 8.7 | 40.7 ± 6.6 | 28 mo |
| **Ding et al. 2022 [XLIX]** | RCT-SC | 102 | NA | 28 mm Arctic Front Advance | NA | 60.9 ± 7.89 | 102 (100) | 0 | 1.65 ± 1.38 | 60.91 ± 4.71 | 38.29 ± 3.68 | 36 mo |
| **Dulai et al. 2021 [LXXX]** | RS-SC | 234 | 927 | 28 mm Arctic Front Advance | 1 application 180 s ± bonus applications | 65.3 ± 10.6 | 134 | 100 | 2.1 ± 1.4 | 55.0 ± 11.2 | 39.1 ± 6.1 | 40 ± 9.2 mo |
| **Ekizler et al. 2017 Bonus freeze [XXXV]** | PS-SC | 56 | 217 | 28 mm Arctic Front Advance | 1 application 240 s ± bonus applications | 58 (48-67) | 217 (100) | 0 | 1.1 ± 1.3 | 57 (54-66) | 38 (36-42) | 12 ± 3 mo |
| **Ekizler et al. 2017 No Bonus freeze[XXXV]** | PS-SC | 80 | 312 | 28 mm Arctic Front Advance | 1 application 240 s ± bonus applications | 62 (49-68) | 312 (100) | 0 | 1.1 ± 1.2 | 56 (54-66) | 39 (36-42) | 13 ± 3 mo |
| **Ekrami et al. 2022 [LXVII]** | RS-SC | 650 | NA | Arctic Front Advance Pro | 1 application TTI + 90 s or TTI + 120 s or 180 s or 240 s ± bonus applications | 58 ± 10 | 509 (78) | 140 (22) | 1 ± 1 | 55 (55–55) | 40 ± 6 | 12 (11–21) mo |
| **Brito et al. 2019 [XXI]** | PS-SC | 102 | NA | 28 mm Arctic Front Advance | 1 applications 240 s | 57.1 ± 6.3 | 102 (100) | 0 | 1 | 62.3 ±6.9 | NA | 19 ± 3 mo |
| **Giannopoulos et al. 2019 [LX]** | RCT-SC | 80 | 319 | 28 mm Arctic Front Advance | 1 application 180-240 s | 61 (55‐67) | 80 (100) | 0 | 1 (1, 2) | 60 (54‐65) | 40.0 (36.0‐43.0) | 6 mo |
| **Glowniak et al. 2019 [LXXXIII]** | PS-SC | 24 | NA | 28 mm Arctic Front Advance Pro | 1 application 180-240 s ± bonus applications | 59.7 ±10.3 | 22 (92) | 2 (8) | NA | 63.2 ± 5.2 | 42.5 ±2.6 | NA |
| **Gunawardene et al. 2018 [LIII]** | RCT-SC | 30 | 119 | 28 mm Arctic Front Advance | 1 application 240 s | 62.0 ± 9.5 | 30 (100) | 0 | 2 (0.25–3) | 59.8 ± 4.5 | NA | 309.7 ± 61.8 d |
| **Hara et al. 2020 [XXVI]** | PS-SC | 186 | 736 | 28 mm Arctic Front Advance | 1 applications 180 s | 62.4 ± 10.9 | 186 (100) | 0 | 1.7 ± 1.3 | 66.6 ± 6.6 | 37.5 ± 5.3 | 45 (30–51) mo |
| **Hasegawa et al. 2019 Pressure guided [LV]** | RCT-SC | 30 | 120 | 28 mm Arctic Front Advance | 2 applications 180 s | 65.7 ± 11.5 | 60 (100) | 0 | 2.1 ± 1.5 | 69.1 ± 6.7 | 36.1 ± 4.9 | 24 mo |
| **Hasegawa et al. 2019 Conventional [LV]** | RCT-SC | 30 | 120 | 28 mm Arctic Front Advance | 2 applications 180 s | 65.7 ± 11.5 | 60 (100) | 0 | 2.1 ± 1.5 | 69.1 ± 6.7 | 36.1 ± 4.9 | 24 mo |
| **Heeger et al. 2019 [XXVII]** | PS-SC | 110 (cb2 55; cb3 55) | 435 | Arctic Front Advance or Arctic Front Advance ST | TTE + 120 s or 180 s. | 62.1 ± 11.4 | 71 (65) | 39 (34) | 2.0 (1.0, 2.0) | NA | NA | 409 (378–421) d |
| **Heeger et al. 2020 [LXXIII]** | RS-DC | 139 | 415 | 28 mm Arctic Front Advance | 2 applications 240 s | 62 ± 11 | 105 (76) | 34 (24) | 1.8 ± 1.4 | NA | 43 ± 6 | 60 (46-72) mo |
| **Heeger et al. 2021 [XXV]** | PS-SC | 300 | 594 4° 594 2° 1188 | 28 mm Arctic Front Advance or Arctic Front Advance Pro | 1 application 180-240 s ± bonus applications | 67 ± 11 | 135 | 165 | 2.0 ± 1.0 | NA | NA | NA |
| **Heeger et al. 2022 [LXIX]** | RS-MC | 317 | 1256 | PolarX cryoballoon | 1 application 180 s ± bonus applications | 64 ± 12 | 209 (66) | 108 (34) | 2.0 ± 1.6 | NA | 36 ± 8 | 226 ± 115 d |
| **Iacopino et al. 2019 2° gen Cryo [XLVII]** | PS-MC | 120 | NA | 28 mm and 23 mm Arctic Front Advance | Operator preference | 60.4 ± 10.0 | 89 | 31 | NA | 57.4 ± 7.4 | 41.7 ± 8.9 | NA |
| **Iacopino et al. 2019 3° gen Cryo [XLVII]** | PS-MC | 120 | NA | 28 mm and 23 mm Arctic Front Advance ST | Operator preference | 60.9 ± 11.2 | 93 | 27 | NA | 58.6 ± 5.9 | 41.2 ± 5.9 | NA |
| **Iacopino et al. 2019 4° gen Cryo [XLVII]** | PS-MC | 120 | NA | 28 mm and 23 mm Arctic Front Advance Pro | Operator preference | 62.4 ± 9.4 | 83 | 37 | NA | 58.4 ± 5.9 | 38.8 ± 8.9 | NA |
| **Jain et al. 2023 [LXX]** | RS-SC | 127 | NA | Arctic Front Advance AND Arctic Front Advance Pro | 1 application 240 s ± bonus applications | 57.7 ± 11.9 | 127 (100) | 0 | NA | 60.1 ± 5.1 | 37.8 ± 5.3 | 6 mo |
| **Jourda et al. 2015 [XXXII]** | PS-SC | 75 | 284 | 28 mm Arctic Front Advance | 2 applications 240 s + bonus applications | 59.9 ± 10.6 | 75 (100) | 0 | 1.3 ± 1.3 | 64.4 ± 7.4 | NA | 12 mo |
| **Kardos et al. 2016 [LXXIX]** | RS-SC | 40 | 157 | 28 mm Arctic Front Advance | 1 application 240 s ± bonus applications | 59 ± 10 | 40 (100) | 0 | NA | NA | 41.3 ±4 | 24 mo |
| **Keçe et al. 2019 TTI 90s [LVII]** | RCT-SC | 25 | 100 | 28 mm and 23 mm Arctic Front Advance | 1 application guided by TTI ± bonus applications | 61 ± 11 | 25 (100) | 0 | 1.6 ± 1.4 | NA | 39 ± 6 | 12 mo |
| **Keçe et al. 2019**  **TTI 120s [LVII]** | RCT-SC | 25 | 99 | 28 mm and 23 mm Arctic Front Advance | 1 application guided by TTI ± bonus applications | 59 ± 11 | 25 (100) | 0 | 1.1 ± 1.0 | NA | 38 ± 5 | 12 mo |
| **Keçe et al. 2019**  **TTI 150s [LVII]** | RCT-SC | 25 | 100 | 28 mm and 23 mm Arctic Front Advance | 1 application guided by TTI ± bonus applications | 60 ± 11 | 25 (100) | 0 | 1.4 ± 1.1 | NA | 40 ± 5 | 12 mo |
| **Knecth et al. 2022 POLARx [LXVIII]** | RS-MC | 40 | NA | 28 mm short tip POLARx | 1 application 180-240 s | 65 ± 11 | 23 (58) | 17 (42) | NA | 60 (54–65) | 40 ± 6 | 12 mo |
| **Knecth et al. 2022 2° gen Cryo [LXVIII]** | RS-MC | 40 | NA | 28 mm Arctic Front Advance Pro | 1 application 180-240 s | 66 ± 9 | 28 (70) | 12 (30) | NA | 60 (55–64) | 40 ± 6 | 12 mo |
| **Knight et al. 2019 [XLIII]** | PS-MC | 344 | 1350 | 28 mm and 23 mm Arctic Front Advance | At least two applications for 180-240 s | 60.2 ± 10.4 | 344(100) | 0 | NA | NA | 39.8 ± 5.6 | NA |
| **Kobori et al. 2022 [LXV]** | RS-SC | 253 | NA | 28 mm Arctic Front Advance or Arctic Front Advance Pro | 1 application 180 s | 70.0 ± 9.6 | 0 | 253 (100) | NA | 58.9 ± 7.9 | 41.4 ± 5.2 | 25.5 ± 12.5 mo |
| **Kumar et al. 2014 [XXIV]** | PS-SC | 90 | 358 | 28 mm and 23 mm Arctic Front | 2 application 240 s ± bonus applications | 57.4 ± 9.5 | 79 | 11 | NA | 56 ± 6 | NA | 397 ± 47 mo |
| **Martins et al. 2018 [LXXV]** | RS-MC | 497 | NA | 28 mm Arctic Front Advance | NA | 59.4 ± 10.6 | 497 (100) | 0 | 1.4 ± 1.5 | NA | NA | 12 mo |
| **Matsuda et al. 2022 [LXIV]** | RS-SC | 388 | NA | 28 mm Arctic Front Advance | 1 application 180 s | 70 ± 11 | 348 (90) | 40 (10) | 2.6 ± 1.4 | 65 ± 10 | 38 ± 6 | 12 mo |
| **Matta et al. 2018 [XXXIV]** | PS-SC | 46 | 181 | 28 mm Arctic Front Advance | 1 application 180 s ± bonus applications | 59 ± 9 | 46 (100) | 0 | NA | 61 (5) | NA | 12 ± 5 mo |
| **Michaelsen et al. 2022 PAF [XLII]** | PS-MC | 520 | NA | 28 mm Arctic Front Advance or Arctic Front Advance Pro | NA | 64.5 ± 13.5 | 520 (100) | 0 | NA | NA | NA | 12 mo |
| **Michaelsen et al. 2022 PersAF [XLII]** | PS-MC | 423 | NA | 28 mm Arctic Front Advance or Arctic Front Advance Pro | NA | 66.0 ± 11.2 | 0 | 423 (100) | NA | NA | NA | 12 mo |
| **Mililis et al. 2023 [LI]** | RCT-SC | 66 | NA | Arctic Front Advance Pro | 1 application 240 s ± bonus applications | 62.74 ± 9.09 | 0 | 66 (100) | 1.71 ± 1.37 | 55.70 ± 6.16 | 43.55 ± 4.74 | 12 mo |
| **Miyamoto et al. 2019 Bonus freeze [LXXXIV]** | RCT-MC | 55 | 438 | 28 mm Arctic Front Advance | 1 application 180 s ± bonus applications | 64.0 ± 11.0 | 55(100) | 0 | 1.9 ± 1.3 | 60 ± 8 | 38 ± 6 | 12 mo |
| **Miyamoto et al. 2019 No Bonus freeze [LXXXIV]** | RCT-MC | 55 | NA | 28 mm Arctic Front Advance | 1 application 180 s ± bonus applications | 63.1 ± 11.8 | 55(100) | 0 | 1.8 ± 1.4 | 61 ± 6 | 37 ± 7 | 12 mo |
| **Molenaar et al. 2018 Short group [LVIII]** | RCT-DB | 74 | 880 | 28 mm and 23 mm Arctic Front Advance or Arctic Front Advance ST | 2 applications 60 ± bonus applications | 58 ± 10 | 74 (100) | 0 | 1.3 ± 1.2 | NA | NA | NA |
| **Molenaar et al. 2018 Medium group [LVIII]** | RCT-DB | 74 | NA | 28 mm and 23 mm Arctic Front Advance or Arctic Front Advance ST | 2 applications 120 ± bonus applications | 59 ±9 | 74 (100) | 0 | 1.2 ± 1.2 | NA | NA | NA |
| **Molenaar et al. 2018 Long group [LVIII]** | RCT-DB | 74 | NA | 28 mm and 23 mm Arctic Front Advance or Arctic Front Advance ST | 2 applications 180 ± bonus applications | 57 ± 9 | 74 (100) | 0 | 1.0 ± 1.0 | NA | NA | NA |
| **Mörtsell et al. 2018 [LVI]** | RCT-SC | 69 single cryo + 70 routine | 269 e 271 | 28 mm Arctic Front Advance | 1 application guided by TTI or 2 applications > 120 s | 61.9 ± 9.08 / 68.3 ± 10.0 | 34+28 | 28+42 | 1.4 ± 1.1 / 1.4 ± 1.1 | NA | NA | 12 mo |
| **Mörtsell et al. 2019 [LXXXII]** | Registry | 982 | NA | 28 and 23 mm Arctic Front Advance Pro | NA | 59.9 ± 10.5 | 740 (75) | 241 (25) | NA | NA | NA | 12 mo |
| **Mugnai et al. 2015 [XXXVIII]** | PS-SC | 500 | NA | 28 mm Arctic Front Advance | 1 application ≥ 180 s | 57.6 ± 12.9 | 427 (85) | 73 (15) | 1.4 ± 1.3 | 58.8 ± 6.9 | 41.6 ± 6.9 | 7.5 ± 4.3 mo |
| **Nitta et al. 2022 [XXXIX]** | PSM-RS-SC | 257 | NA | 28 mm Arctic Front Advance | 1 application 180-240 s | 63.4 ± 10.3 | 0 | 257 (100) | 1.94 ± 1.44 | 59.8 ± 12.3 | 41.0 ± 6.0 | 615 d |
| **Reddy et al. 2014 [XLVI]** | PS-MC | 21 | 84 | 28 mm Arctic Front Advance Pro | At least 1 application 240 s + bonus applications | 60 ± 11 | 21(100) | 0 | NA | NA | NA | 3.4 (2.9–4.1) mo |
| **Ruiz et al. 2019 [LXXVIII]** | RS-SC | 172 | 671 | 28 mm and 23 mm Arctic Front Advance | 1 application 180-240 s ± bonus applications | 58.1 ± 10.5 | 134 (78) | 38 (22) | NA | NA | NA | 27 (14-41) mo |
| **Seidl et al. 2021 2° gen Cryo RCT [XXXI]** | RCT-SC | 56 | NA | 28 mm Arctic Front Advance | At least 2 application 240 s ± bonus applications | 66 ± 11 | 56 (100) | 0 | 2 (0–6) | 61± 6 | NA | 13.2 ± 3.6 mo |
| **Seidl et al. 2021 2° gen Cryo Registry [XXXI]** | PS-SC | 22 | 89 | 28 mm Arctic Front Advance | At least 2 application 240 s ± bonus applications | 67 ± 18 | 22 (100) | 0 | 3 (0–6) | 60 (53–72) | NA | 12 mo |
| **Seki et al. 2020 [XVI]** | PSM-RS-SC | 50 | 193 | 28 mm Arctic Front Advance or Arctic Front Advance Pro | 1 application TTI + 90 s or TTI + 120 s or 180 s ± bonus applications | 66 ± 13 | 41 (82) | 9 (18) | 2 (1-3) | 58 ± 11 | 40 ± 6 | 21 ± 6 mo |
| **Sharma et al. 2017 [XXXVI]** | PS-SC | 122 | NA | 28 mm Arctic Front Advance | 2 applications 180 s | 62.7 ± 10.1 | 104 (85) | 18 (15) | 2.1 ± 1.5 | 58.2 ± 10.0 | NA | 12 mo |
| **Shi et al. 2021 [L]** | RCT-MC | 52 | NA | 28 mm Arctic Front Advance | 2 application 240 s ± bonus applications | 62.4 ± 8.4 | 0 | 52 (100) | NA | 56.0 ± 7.2 | 46 ± 6 | 12 mo |
| **Straube et al. 2016 [XXXIII]** | PS-SC | 173 | 673 | 28 mm and 23 mm Arctic Front Advance | At least two applications for 180-240 s + bonus applications | 64 ± 10 | 0 | 173 (100) | NA | 54 ± 8 | 46 ± 7 | 14 (12–19) mo |
| **Tanese et al. 2023 [XL]** | PS-MC | 137 | NA | PolarX cryoballoon | 1 application 180-240 s ± bonus applications | 63.3 ± 10.7 | 137 (100) | 0 | NA | NA | NA | 12 mo |
| **Tebbenjohanns et al. 2016 No Bonus freeze [XXX]** | PS-SC | 53 | 211 | 28 mm Arctic Front Advance | 2 applications 240 s | 66 ± 10 | 38 (72) | 15 (28) | 1.8 ± 1.2 | NA | 40 ± 6 | 458 ± 107 d |
| **Tebbenjohanns et al. 2016 Bonus freeze [XXX]** | PS-SC | 139 | NA | 28 mm Arctic Front Advance | 2 applications 240 s | 61 ± 11 | 87 (63) | 51 (37) | 1.9 ± 1.3 | NA | 41 ± 7 | 458 ± 107 d |
| **Tokuda et al. 2019 [XVIII]** | PS-SC | 230 | 920? | 28 mm Arctic Front Advance | Up to 3 applications 180 s ± bonus RF applications | 58.9 ± 9.6 | 230 (100) | 0 | NA | 63.6 ± 5.0 | 24.0 ± 3.2 | 20.1 ± 12.7 mo |
| **Tscholl et al. 2018 [LXXVII]** | RS-SC | 80 | NA | 28 mm Arctic Front Advance | 1 application 240 s ± bonus applications | 74.5 (65-77) | 37 (46) | 43 (54) | 4 (2-5) | 65 (60-69) | NA | 12 (6-24) mo |
| **Urbanek et al. 2023 [IX]** | RS-SC | 200 | 783 | 28 mm Arctic Front Advance | 1 application 240 s ± bonus applications | 68 (58–77) | 127 (64) | 73 (26) | 3 (1–4) | NA | 40 (36–44) | 392 (190–513) d |
| **Vaishnav et al. 2019 [LXXI]** | RS-SC | 150 | NA | 28 mm Arctic Front Advance | 2 applications of 180 and 120 s | 64.45 ± 9.71 | 86 (58) | 64 (42) | 2.36 ± 1.61 | 55.90 ± 8.08 | NA | 15.56 ± 9.42 mo |
| **Watanabe et al. 2018 [LXII]** | RCT-SC | 25 | 100 | 28 mm Arctic Front Advance | 1 application 180 s ± bonus applications | 62 ± 1 | 25(100) | 0 | NA | 63 ± 5 | 39±6 | 12 mo |
| **Wei et al. 2022 [XLI]** | PS-MC | 247 | 976 | Arctic Front Advance Pro | 1 application TTI + 90 s or TTI + 120 s or 180 s ± bonus applications | 60.8 ± 10.0 | 247 (100) | 0 | 1.6 ± 1.2 | 65.9 ± 5.4 | 38.7 ± 3.9 | 801 d |
| **Wieczorek et al. 2019 [XXVIII]** | PS-SC | 100 | 390 | 28 mm and 23 mm Arctic Front Advance | 1 application 180 s ± bonus applications | 58 ± 12 | 61 | 39 | NA | 54 ± 7 | NA | 9 mo |
| **Xia et al. 2020 [LXXII]** | RS-SC | 369 | NA | 28 mm Arctic Front Advance | 1 application 180 s | 59.0 ± 9.8 | 271 (73) | 98 (27) | 2.1 ± 1.5 | 63.1 ± 4.9 | 39.0 ± 5.0 | 615 (415-822.5) d |
| **Yokokawa et al. 2017 [XXIX]** | PS-SC | 71 | NA | 28 mm Arctic Front Advance | 1 application 180-240 s | 63 ± 10 | 71 (100) | 0 | 1.3 ± 1.1 (0–4) | 0.59 ± 0.06 | 42 ± 6 | 25 ± 5 mo |
| **Zhao et al. 2017 [XXII]** | PS-SC | 50 | NA | 23 mm and 28 mm Arctic Front Advance | 1 application 240 s ± bonus applications | 60.4  ±  11.2 | 50 (100) | 0 | 0.82  ±  0.42 | 61  ±  7.9 | NA | 24 mo |
| **Maurhofer et al. 2023 [XC]** | PSM-SC | 80 | NA | 23 mm and 28 mm Arctic Front Advance | 1 application 180 s | 62.2 (54.7- 70.7) | 80(100) | 0 | NA | 60.0 (55.8 - 65.0) | 42 (38- 45) | 12.8 (12.2–13.1) mo |

***AF:*** *Atrial Fibrillation;* ***d:*** *day;* ***DC:*** *Double Center;* ***NA:*** *not available;* ***LVEF:*** *Left Ventricular Ejection Fraction;* ***mo:*** *months;* ***MC:*** *Multicenter;* ***PAF:*** *Paroxysmal Atrial Fibrillation;* ***PerAF****: Persistent Atrial Fibrillation;* ***PS:*** *Prospective Study;* ***RCT:*** *Randomized Clinical Trial;* ***RS:*** *Retrospective Study;* ***SC:*** *Single Center;* ***TTI:*** *Time To Isolation***;**

*References in brackets are reported in Supplementary Materials.*

**Supplemental Table 3. Pulmonary Vein Isolation Confirmation Methods**

| **Study** | **Pulmonary Vein Isolation Confirmation Method** |
| --- | --- |
| **Badertscher et al. 2023 [VIII]** | High Density mapping with or without Entrance and Exit block with basket shape Farapulse catheter |
| **Bohnen et al. 2022 [VII]** | Entrance and Exit block with Lasso catheter. |
| **Davong et al. 2023 [X]** | Entrance and Exit block with basket shape Farapulse catheter |
| **Füting et al. 2022 [VI]** | High Density mapping with or without Entrance and Exit block |
| **Gunawardene et al. 2022 (PVI) [V]** | High Density mapping |
| **Kueffer et al. 2023 [IV]** | High Density mapping or with Entrance and Exit block with basket shape Farapulse catheter |
| **Lemoine et al. 2022 [XIII]** | High Density mapping with or without Entrance block |
| **Magni et al. 2022 [III]** | Disappearance of PV potentials on Farapulse catheter and Exit block with basket shape Farapulse catheter |
| **Reddy et al. 2020 [I]** | High Density mapping |
| **Reddy et al. 2021 [XII]** | High Density mapping with Entrance and Exit block with Lasso catheter |
| **Reddy et al. 2023 [XV]** | Entrance block with basket shape Farapulse catheter |
| **Ruwald et al. 2023 [XI]** | Entrance and Exit block with basket shape Farapulse catheter |
| **Tilz et al. 2023 [II]** | Entrance and Exit block with Lasso catheter |
| **Urbanek et al. 2023 [IX]** | Entrance block with basket shape Farapulse catheter |
| **Della Rocca et al. 2024 [LXXXV]** | Entrance block with basket shape Farapulse catheter |
| **Badertscher et al. 2024 [LXXXVI]** | High Density mapping |
| **Lee et al. 2023 [LXXXVII]** | Entrance block with basket shape Farapulse catheter |
| **Meininghaus et al. 2023 [LXXXVIII]** | Entrance and Exit block with basket shape Farapulse catheter |
| **Wörmann et al. 2023 [LXXXIX]** | Entrance and Exit block with basket shape Farapulse catheter |

*References in brackets are reported in Supplementary Materials.*

**Supplemental Table 4. Pooled rate of Periprocedural Complications for Pulsed Field Ablation and Cryoballoon Ablation.**

| **Complications** | **Pulsed Field Ablation** | **Cryoballoon Ablation** | **p- value** |
| --- | --- | --- | --- |
| **Coronary spasm or ST-elevation due to air embolism** | 0.85% (95%CI: 0.00 - 2.2) | 0.33% (95%CI: 0.03 - 0.62) | 0.40 |
| **Esophageal lesion** | 0.00% (95%CI: 0.00 - 0.00) | 0.15% (95%CI: 0.04 - 0.23) | 0.87 |
| **Stroke and/or Transient Ischemic Attack** | 0.49% (95%CI: 0.00 - 1.01) | 0.45% (95%CI: 0.29 - 0.62) | 0.58 |
| **Pericardial tamponade or effusion with need for pericardiocentesis** | 0.80% (95%CI: 0.28 - 1.33) | 0.40% (95%CI: 0.24 - 0.56) | 0.12 |
| **Phrenic nerve palsy** | 0.01% (95%CI: 0.00 - 0.07) | 1.84% (95%CI: 1.58 - 2.10) | <0.001 |
| **Transient phrenic nerve palsy** | 0.01% (95%CI: 0.00 - 0.05) | 1.60% (95%CI: 1.21- 2.00) | <0.001 |
| **Persistent phrenic nerve palsy** | 0.00% (95%CI: 0.00 - 0.02) | 0.30% (95%CI: 0.01 - 1.23) | 0.17 |
| **Death** | 0.00% (95%CI: 0.00 - 0.01) | 0.00% (95%CI: 0.00 - 0.00) | 0.79 |
| **Bleeding/vascular complications** | 1.30% (95%CI: 0.60 - 1.90) | 1.20% (95%CI: 0.90 - 1.50) | 0.36 |
| **Acute Kidney Injury** | 0.00% (95%CI: 0.00 - 0.01) | 0.00% (95%CI: 0.00 - 0.01) | 1.00 |

***CI:*** *Confidence Interval*

**Supplemental Table 5. Phrenic nerve capture monitoring during Right pulmonary veins ablation.**

| **Study** | **Phrenic nerve capture monitoring during RPVs ablation** |
| --- | --- |
| **Abdin et al. 2019 [LXXXI]** | Fluoroscopy and/or tactile feedback of diaphragmatic contraction and compound motor action potentials. |
| **Ahn et al. 2023 [LII]** | Fluoroscopy and/or tactile feedback of diaphragmatic contraction. |
| **Akhtar et al. 2021 [LXIII]** | Fluoroscopy and/or tactile feedback of diaphragmatic contraction. |
| **Akkaya et al. 2017 [XXIII]** | Fluoroscopy and/or tactile feedback of diaphragmatic contraction and compound motor action potentials. |
| **Ali et al. 2021 [XLIV]** | Fluoroscopy and/or tactile feedback of diaphragmatic contraction. |
| **Andrade et al. 2019 [LXI]** | Fluoroscopy and/or tactile feedback of diaphragmatic contraction. |
| **Aryana et al. 2016 [XLV]** | Fluoroscopy and/or tactile feedback of diaphragmatic contraction. |
| **Baimbetov et al. 2022 [XLVIII]** | Fluoroscopy and/or tactile feedback of diaphragmatic contraction. |
| **Buist et al. 2017 [LIV]** | Fluoroscopy and/or tactile feedback of diaphragmatic contraction. |
| **Canpolat et al. 2019 [XIX]** | Fluoroscopy and/or tactile feedback of diaphragmatic contraction. |
| **Chen et al. 2019 [LXXVI]** | Fluoroscopy and/or tactile feedback of diaphragmatic contraction. |
| **Choi et al. 2022 [LXVI]** | Fluoroscopy and/or tactile feedback of diaphragmatic contraction. |
| **Chun et al. 2016 [LIX]** | Fluoroscopy and/or tactile feedback of diaphragmatic contraction. |
| **Curnis et al. 2017 [XVII]** | Fluoroscopy and/or tactile feedback of diaphragmatic contraction. |
| **Davies et al. 2020 [LXXIV]** | Fluoroscopy and/or tactile feedback of diaphragmatic contraction. |
| **Ding et al. 2022 [XLIX]** | Fluoroscopy and/or tactile feedback of diaphragmatic contraction. |
| **Dulai et al. 2021 [LXXX]** | Fluoroscopy and/or tactile feedback of diaphragmatic contraction. |
| **Ekizler et al. 2017 [XXXV]** | Fluoroscopy and/or tactile feedback of diaphragmatic contraction. |
| **Ekrami et al. 2022 [LXVII]** | Fluoroscopy and/or tactile feedback of diaphragmatic contraction. |
| **Glowniak et al. 2019 [LXXXIII]** | Fluoroscopy and/or tactile feedback of diaphragmatic contraction. |
| **Gunawardene et al. 2018 [LIII]** | Fluoroscopy and/or tactile feedback of diaphragmatic contraction and compound motor action potentials. |
| **Hara et al. 2020 [XXVI]** | Fluoroscopy and/or tactile feedback of diaphragmatic contraction and compound motor action potentials. |
| **Heeger et al. 2019 [XXVII]** | Fluoroscopy and/or tactile feedback of diaphragmatic contraction and compound motor action potentials. |
| **Heeger et al. 2020 [LXXIII]** | Fluoroscopy and/or tactile feedback of diaphragmatic contraction and compound motor action potentials. |
| **Heeger et al. 2021 [XXV]** | Fluoroscopy and/or tactile feedback of diaphragmatic contraction and compound motor action potentials. |
| **Heeger et al. 2022 [LXIX]** | Fluoroscopy and/or tactile feedback of diaphragmatic contraction, compound motor action potentials and diaphragm movement sensor. |
| **Iacopino et al. 2019 [XLVII]** | Fluoroscopy and/or tactile feedback of diaphragmatic contraction. |
| **Jain et al. 2023 [LXX]** | Fluoroscopy and/or tactile feedback of diaphragmatic contraction. |
| **Jourda et al. 2015 [XXXII]** | Fluoroscopy and/or tactile feedback of diaphragmatic contraction. |
| **Kardos et al. 2016 [LXXIX]** | Fluoroscopy and/or tactile feedback of diaphragmatic contraction. |
| **Keçe et al. 2019 [LVII]** | Fluoroscopy and/or tactile feedback of diaphragmatic contraction. |
| **Knecth et al. 2022 [LXVIII]** | Fluoroscopy and/or tactile feedback of diaphragmatic contraction. |
| **Knight et al. 2019 [XLIII]** | Fluoroscopy and/or tactile feedback of diaphragmatic contraction and compound motor action potentials. |
| **Kobori et al. 2022 [LXV]** | Fluoroscopy and/or tactile feedback of diaphragmatic contraction and compound motor action potentials. |
| **Kumar et al. 2014 [XXIV]** | Fluoroscopy and/or tactile feedback of diaphragmatic contraction. |
| **Matsuda et al. 2022 [LXIV]** | Fluoroscopy and/or tactile feedback of diaphragmatic contraction. |
| **Matta et al. 2018 [XXXIV]** | Fluoroscopy and/or tactile feedback of diaphragmatic contraction. |
| **Mililis et al. 2023 [LI]** | Fluoroscopy and/or tactile feedback of diaphragmatic contraction. |
| **Miyamoto et al. 2019 [LXXXIV]** | Fluoroscopy and/or tactile feedback of diaphragmatic contraction and compound motor action potentials. |
| **Molenaar et al. 2018 [LVIII]** | Fluoroscopy and/or tactile feedback of diaphragmatic contraction. |
| **Mortsell et al. 2018 [LVI]** | Fluoroscopy and/or tactile feedback of diaphragmatic contraction. |
| **Mortsell et al. 2019 [LXXXII]** | Fluoroscopy and/or tactile feedback of diaphragmatic contraction. |
| **Mugnai et al. 2015 [XXXVIII]** | Fluoroscopy and/or tactile feedback of diaphragmatic contraction. |
| **Nitta et al. 2022 [XXXIX]** | Fluoroscopy and/or tactile feedback of diaphragmatic contraction. |
| **Reddy et al. 2014 [XLVI]** | Fluoroscopy and/or tactile feedback of diaphragmatic contraction. |
| **Ruiz et al. 2019 [LXXVIII]** | Fluoroscopy and/or tactile feedback of diaphragmatic contraction. |
| **Seidl et al. 2021 [XXXI]** | Fluoroscopy and/or tactile feedback of diaphragmatic contraction and compound motor action potentials. |
| **Seki et al. 2020 [XVI]** | Fluoroscopy and/or tactile feedback of diaphragmatic contraction. |
| **Shi et al. 2021 [L]** | Fluoroscopy and/or tactile feedback of diaphragmatic contraction and compound motor action potentials. |
| **Straube et al. 2016 [XXXIII]** | Fluoroscopy and/or tactile feedback of diaphragmatic contraction. |
| **Tanese et al. 2023 [XL]** | Fluoroscopy and/or tactile feedback of diaphragmatic contraction and diaphragm movement sensor. |
| **Tebbenjohanns et al. 2016 [XXX]** | Fluoroscopy and/or tactile feedback of diaphragmatic contraction. |
| **Tscholl et al. 2018 [LXXVII]** | Fluoroscopy and/or tactile feedback of diaphragmatic contraction. |
| **Urbanek et al. 2023 [IX]** | Fluoroscopy and/or tactile feedback of diaphragmatic contraction and compound motor action potentials. |
| **Vaishnav et al. 2019 [LXXI]** | Fluoroscopy and/or tactile feedback of diaphragmatic contraction. |
| **Wei et al. 2022 [XLI]** | Fluoroscopy and/or tactile feedback of diaphragmatic contraction. |
| **Wieczorek et al. 2019 [XXVIII]** | Fluoroscopy and/or tactile feedback of diaphragmatic contraction. |
| **Yokokawa et al. 2017 [XXIX]** | Fluoroscopy and/or tactile feedback of diaphragmatic contraction and compound motor action potentials. |
| **Zhao et al. 2017 [XXII]** | Fluoroscopy and/or tactile feedback of diaphragmatic contraction. |
| **Maurhofer et al. 2023 [XC]** | Fluoroscopy and/or tactile feedback of diaphragmatic contraction. |

**Supplemental Table 6. Follow-up Strategy of the Studies Included in the Individual Patient Data Analysis**

| **Study** | **Follow up** |
| --- | --- |
| **Pulsed Field Ablation** | |
| **Schmidt et al. 2023 [XIV]** | Periodical scheduled visits with 12-lead ECG and 24-h/120-h Holter |
| **Lemoine et al. 2022 [XIII]** | Periodical scheduled visits with 12-lead ECG and 24-h Holter |
| **Reddy et al. 2023 [XV]** | Periodical scheduled visits with 12-lead ECG and 24-h Holter and trans-telephonic ECG. |
| **Della Rocca et al. 2024 [LXXXV]** | Periodical scheduled visits with 12-lead ECG and 24-h/7-days Holter |
| **Wörmann et al. 2023 [LXXXVIII]** | Periodical scheduled visits with 12-lead ECG, 48-h Holter and photopletysmogram app‐based tele‐consultation. |
| **Cryoballoon Ablation** | |
| **Ding et al. 2022 [XLIX]** | Periodical scheduled visits with 12-lead ECGs and 24-h Holter and trans-telephonic follow-up. |
| **Tanese et al. 2023 [XL]** | Periodical scheduled visits with 12-lead ECGs and 24-h Holter. |
| **Choi et al. 2022 [LXVI]** | Periodical scheduled visits with 12-lead ECGs and 24-h Holter and portable event monitor ECG. |
| **Wei et al. 2022 [XLI]** | Periodical scheduled visits with 12-lead ECG and 24-h Holter. |
| **Jain et al. 2023 [LXX]** | Periodical scheduled visits with 12-lead ECG and 24-h Holter and portable event recorder monitor. |
| **Curnis et al. 2017 [XVII]** | Periodical scheduled visits with 12-lead ECG and 24-h Holter. |
| **Gunawardene et al. 2018 [LIII]** | Periodical scheduled visits with 12-lead ECG and 5-days Holter. |
| **Tokuda et al. 2019 [XVIII]** | Periodical scheduled visits with 12-lead ECG, 24-h Holter and portable event recorder monitor. |
| **Boghossian et al. 2020 [XX]** | Periodical scheduled visits with 12-lead ECG and 24-h Holter. |
| **Heeger et al. 2020 [LXXIII]** | Periodical scheduled visits with 12-lead ECGs and 24-h Holter and trans-telephonic follow-up. |
| **Davies et al. 2020 [LXXIV]** | 12-lead ECG and 24-h Holter triggered by symptoms. |
| **Zhao et al. 2017 [XXII]** | Periodical scheduled visits with 12-lead ECG and 24-h Holter. |
| **Buist et al. 2017 [LIV]** | Periodical scheduled visits with 12-lead ECG and 24/72-h Holter. |
| **Hasegawa et al. 2019 [LV]** | NA |
| **Kumar et al. 2014 [XXIV]** | NA |
| **Knight et al. 2019 [XLIII]** | Periodical scheduled visits with 12-lead ECG and 24/48-h Holter. |
| **Hara et al. 2020 [XXVI]** | Periodical scheduled visits with 12-lead ECG, 24-h Holter and 14 days external loop recorder. |
| **Ruiz et al. 2019 [LXXVIII]** | Periodical scheduled visits with 12-lead ECG and 24-h Holter. |
| **Ali et al. 2021 [XLIV]** | Periodical scheduled visits with 12-lead ECG and 24-h Holter. |
| **Kardos et al. 2016 [LXXIX]** | Periodical scheduled visits with 12-lead ECG and 24-h Holter. |
| **Jourda et al. 2015 [XXXII]** | Periodical scheduled visits with 12-lead ECG and 24-h Holter. |
| **Urbanek et al. 2023 [IX]** | Periodical scheduled visits with 12-lead ECG and 72-h Holter. |

**Supplemental Table 7. Meta-regression output of Efficacy endpoint in CRYO and PFA patients**

|  | Coeff. (95% CI) | τ^2^ | I^2^ | p-value |
| --- | --- | --- | --- | --- |
| *CRYO* | | | | |
| Age | -0.0002 (-0.001– 0.0009) | 0.0002 | 78.10% | 0.73 |
| Male Sex | 0.00001(-0.0006- 0.0006) | 0.0002 | 79.43% | 0.96 |
| Year of Study publication | -0.002 (-0.003 -0.0002) | 0.0002 | 75.06% | 0.08 |
| Study N. of patients | -0.00002 (-0.00004- 0.000002) | 0.0001 | 75.32% | 0.05 |
| Paroxysmal AF | 0.0001 (-0.00007 -0.0003) | 0.0002 | 78.17% | 0.25 |
| PVI confirmation method | 0.007 (-0.015 -0.029) | 0.001 | 78.94% | 0.52 |
| *PFA* | | | | |
| Age | 0.0002 (-0.0007 - 0.001) | 0.00001 | 0.01% | 0.65 |
| Male Sex | 0.00007 (-0.0002- 0.0003) | 0.00001 | 0.02% | 0.52 |
| Year of Study publication | 0.0003 (-0.004- 0.004) | 0.000001 | 0.02% | 0.89 |
| Study N. of patients | 0.00001 (-0.00002 - 0.00004) | 0.000001 | 0.02% | 0.67 |
| Paroxysmal AF | 0.00002 (-0.0001 -0.0001) | 0.000001 | 0.01% | 0.74 |
| PVI confirmation method | -0.003 (-0.009 -0.004) | 0.000001 | 0.001% | 0.41 |

**Supplemental Table 8. Meta-regression output of Overall complications in CRYO and PFA patients**

|  | Coeff. (95% CI) | τ^2^ | I^2^ | p-value |
| --- | --- | --- | --- | --- |
| *CRYO* | | | | |
| Age | -0.0003 (-0.002– 0.0001) | 0.001 | 85.71% | 0.74 |
| Male Sex | -0.0009(-0.006- 0.02) | 0.001 | 84.51% | 0.07 |
| Year of Study publication | -0.002 (-0.04 -0.002) | 0.001 | 85.75% | 0.31 |
| Paroxysmal AF | 0.0002 (-0.00007 -0.0003) | 0.0002 | 78.17% | 0.25 |
| Study N. of patients | 0.000001 (-0.00002- 0.00005) | 0.002 | 80.21% | 0.74 |
| Bonus applications | 0.007 (-0.015 -0.029) | 0.001 | 85.94% | 0.52 |
| *PFA* | | | | |
| Age | -0.0003 (-0.002 - 0.002) | 0.00001 | 13.72% | 0.78 |
| Male Sex | -0.0002 (-0.001- 0.0009) | 0.00001 | 11.89% | 0.76 |
| Year of Study publication | 0.007 (-0.003- 0.02) | 0.000001 | 2.76% | 0.15 |
| Study N. of patients | -0.01 (-0.00001 - 0.0002) | 0.000001 | 0.01% | 0.08 |
| Bonus applications | 0.006 (-0.014 -0.019) | 0.0001 | 14.34% | 0.62 |
| CRYO vs PFA |  |  |  |  |
| Energy type (PFA vs CRYO) | -0.022 (-0.04 - -0.002) | 0.0009 | 81.00% | **0.03** |

**Supplemental Figure 1: The Farawave^TM^ ablation catheter (A) in flower (B) and basket shape (C).**

**
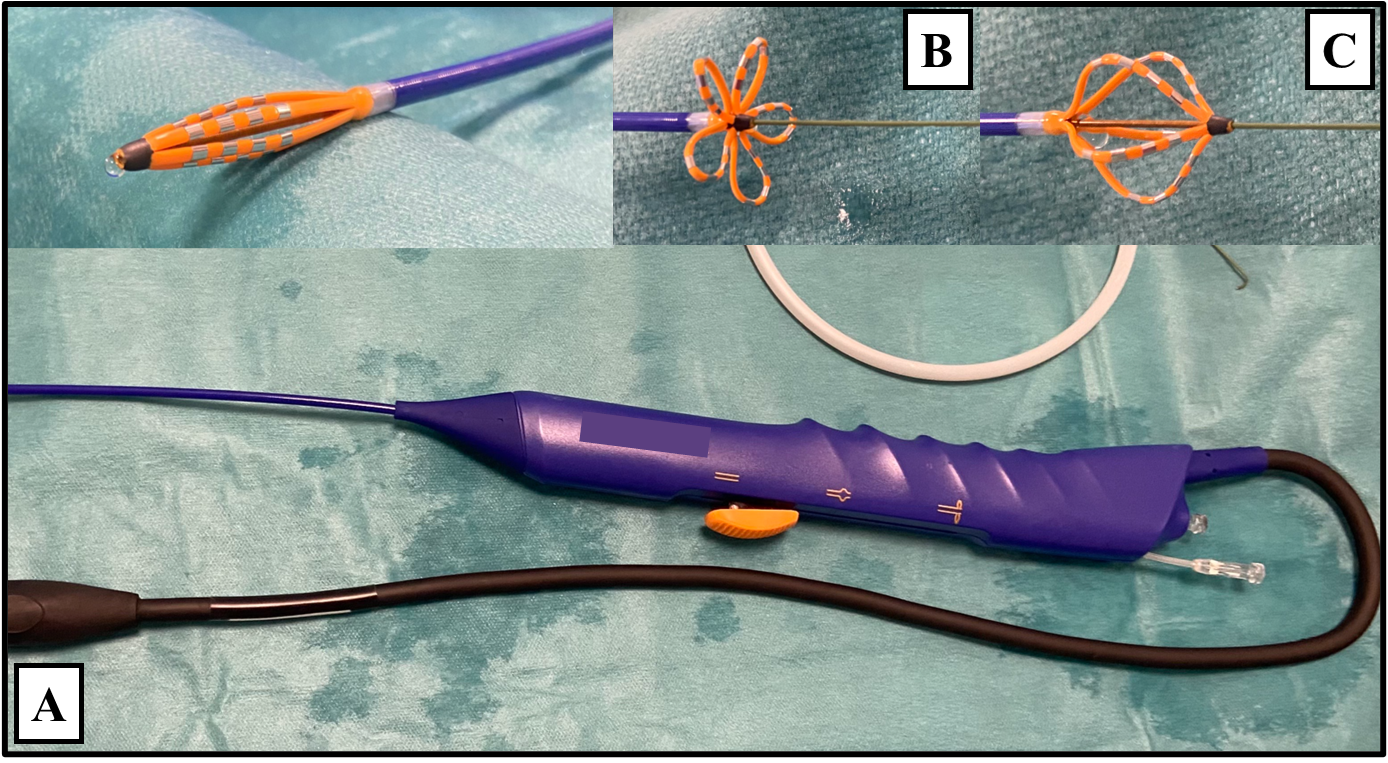
**

**Supplemental Figure 2: Evidence search and selection of Preferred Reporting Items for Systematic Reviews and Meta-Analyses (PRISMA) for Pulsed Field Ablation (A) and Cryoballoon Ablation (B).**

**
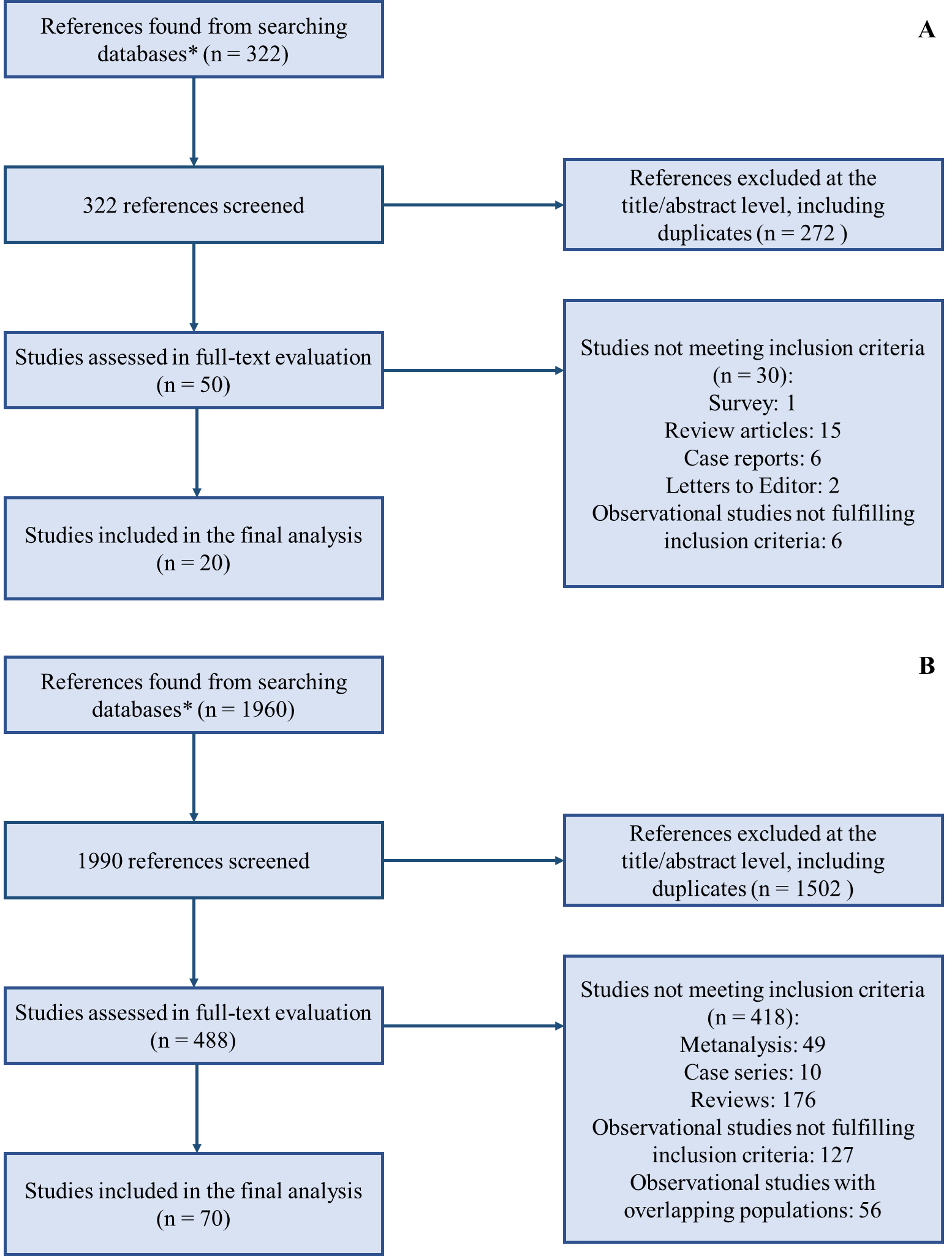
**

** Pubmed, Scopus, Cochrane*

**Supplemental Figure 3. Forest plot comparing Acute Procedural Success per Vein (A), Acute Procedural Success per Patient (B) between Pulsed Field Ablation and Cryoballoon Ablation including only PFA studies without additional lesions in the left atrium.**

***
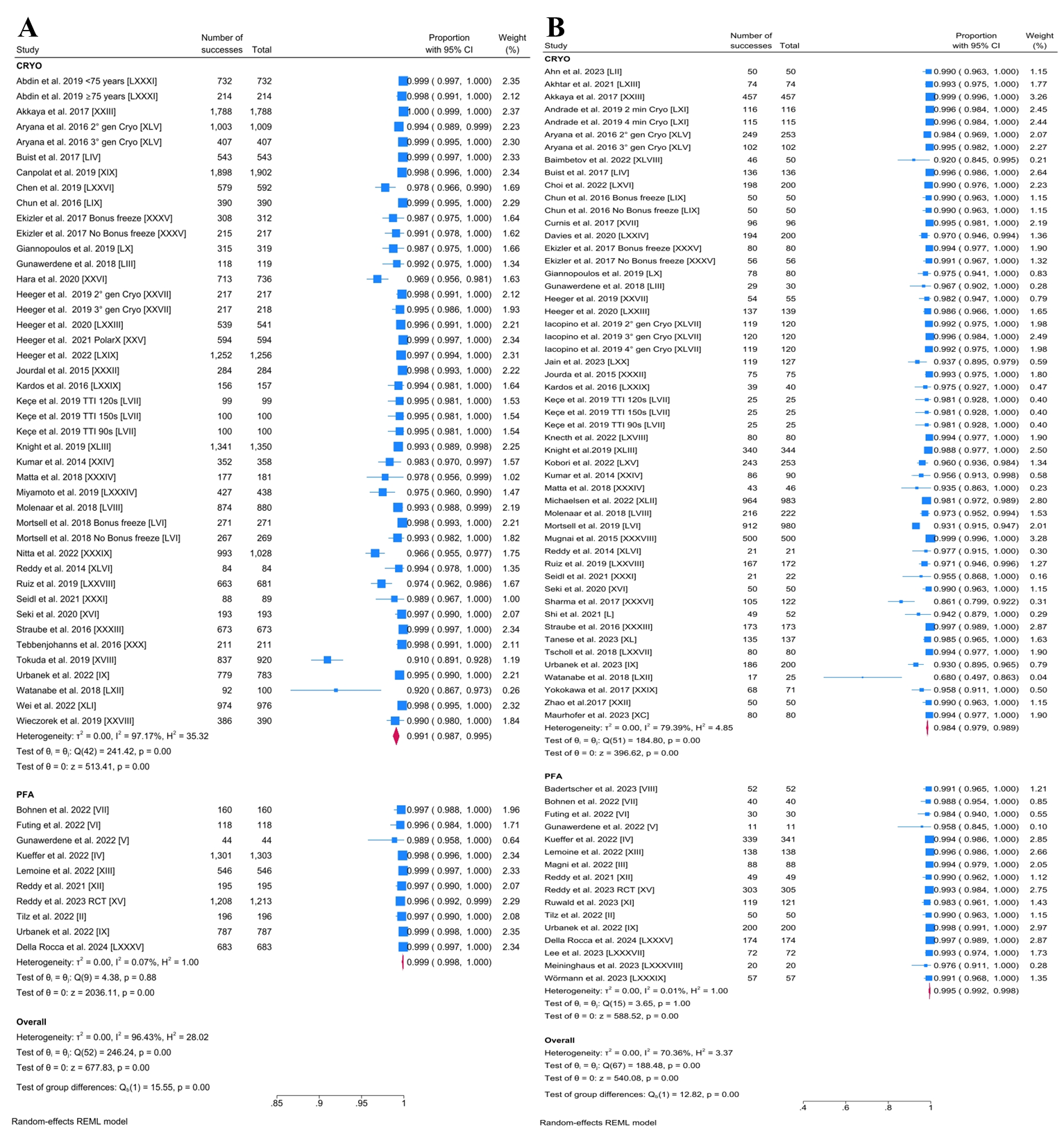
***

**Supplemental Figure 4. Forest plot comparing Acute Procedural Success per Vein (A), Acute Procedural Success per Patient (B) between Pulsed Field Ablation and Cryoballoon Ablation including only PFA studies with only Entrance and Exit block PVI confirmation method.**

**
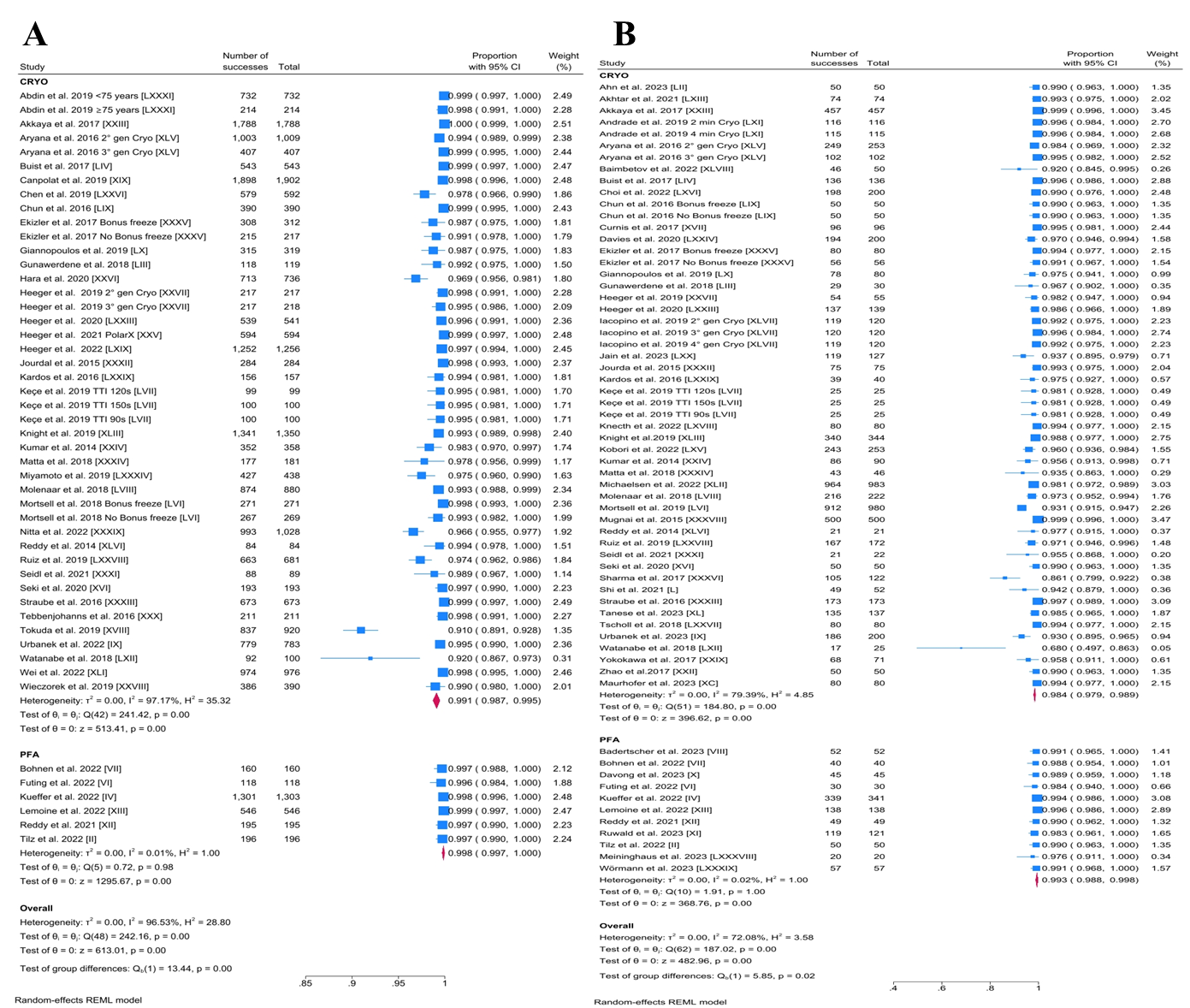
**

**Supplemental Figure 5. Forest plot comparing Acute Procedural Success per Vein (A), Acute Procedural Success per Patient (B) between Pulsed Field Ablation and Cryoballoon Ablation including only studies with 4^th^ generation cryoballoons.**

**
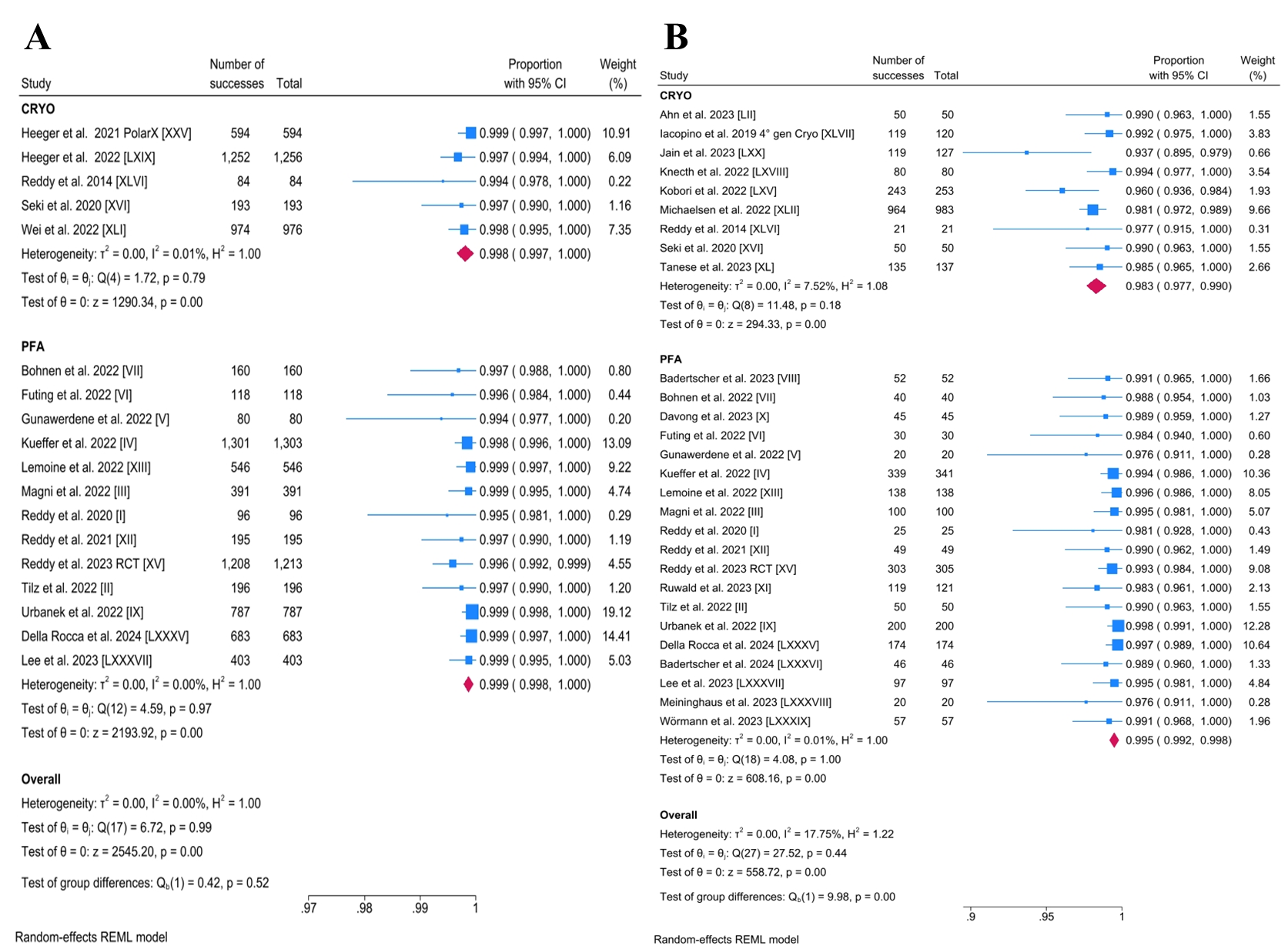
**

**Supplemental Figure 6. Forest plot comparing Acute Procedural Success per Vein (A), Acute Procedural Success per Patient (B), Overall Periprocedural Complications (C) and Major Periprocedural Complications (D) between Pulsed Field Ablation and Cryoballoon Ablation in studies enrolling ≥ 100 patients.**

**
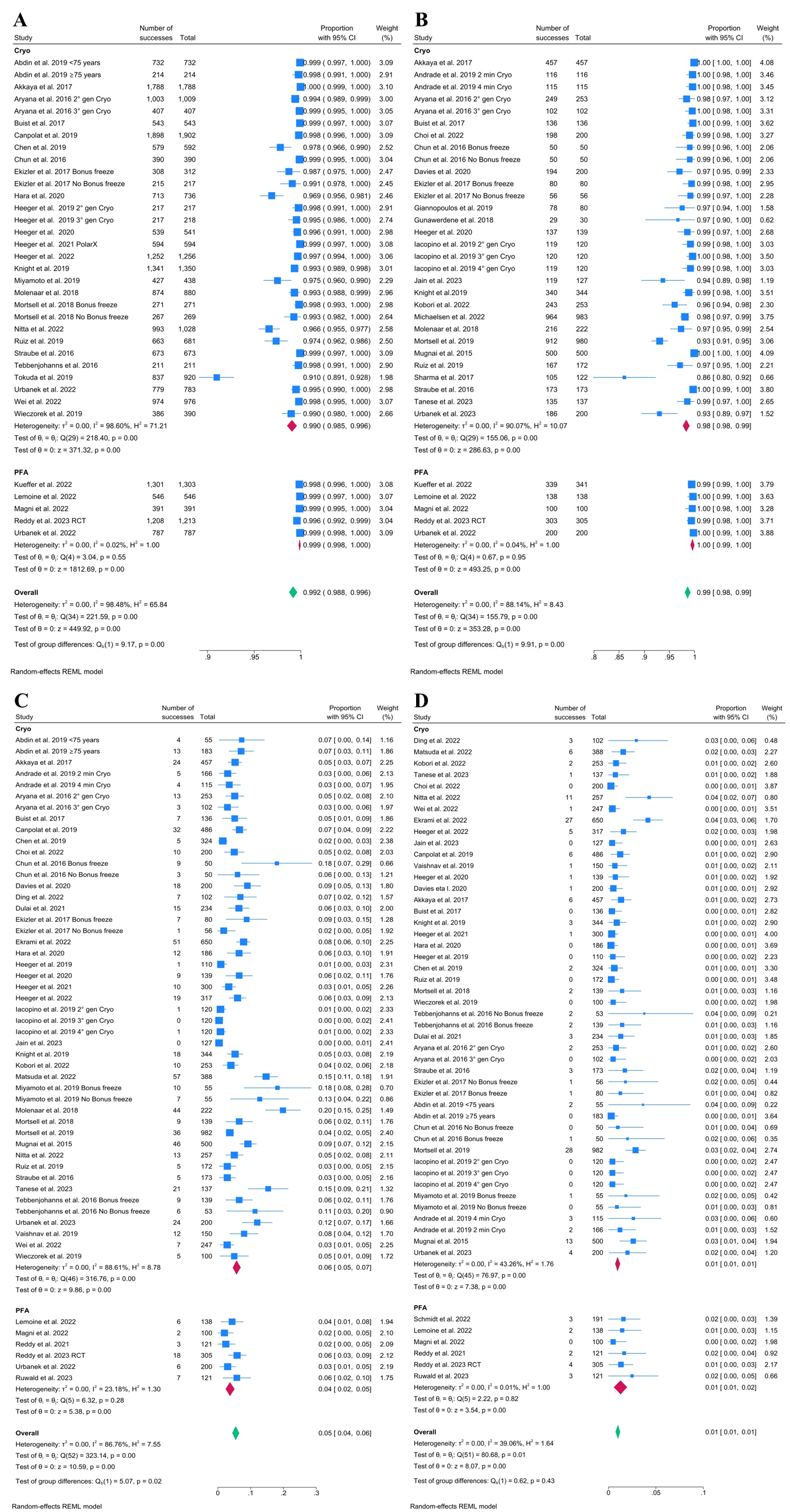
**

**
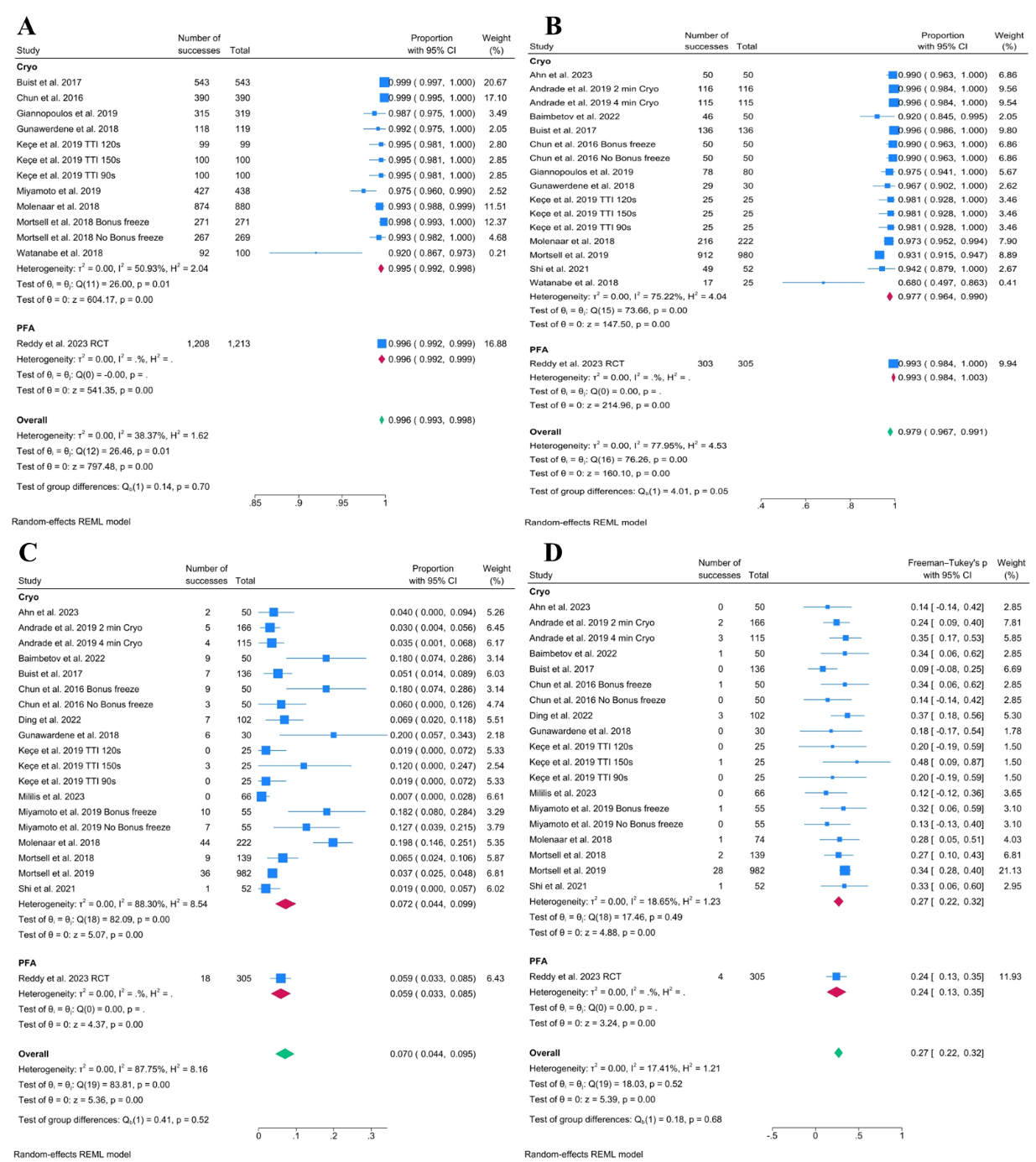
Supplemental Figure 7. Forest plot comparing Acute Procedural Success per Vein (A), Acute Procedural Success per Patient (B), Overall Periprocedural Complications (C) and Major Periprocedural Complications (D) between Pulsed Field Ablation and Cryoballoon Ablation in Randomized Clinical Trials**


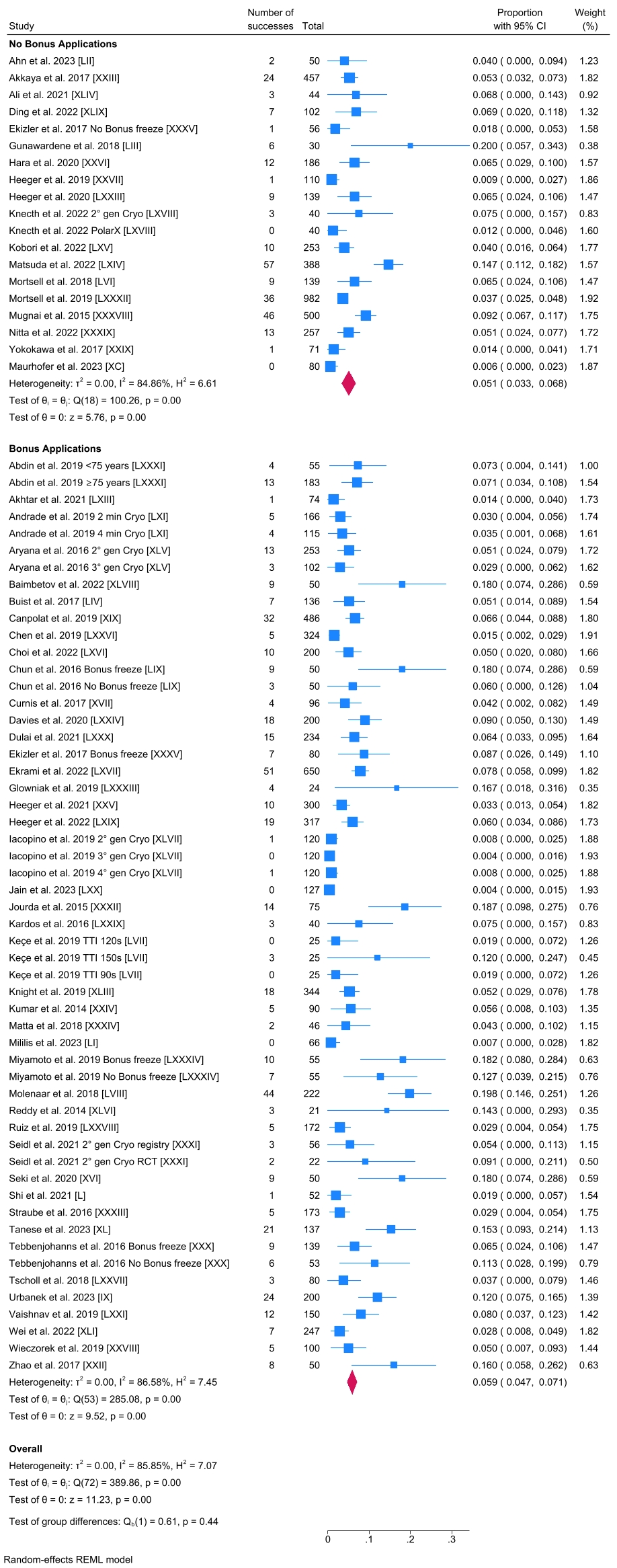
**Supplemental Figure 8. Forest plot comparing Overall complications in CRYO patients between Bonus Applications and No Bonus Applications.**

**Supplemental Figure 9. Forest plot comparing Overall complications between Pulsed Field Ablation and Cryoballoon Ablation including only studies with 4^th^ generation cryoballoons.**

**
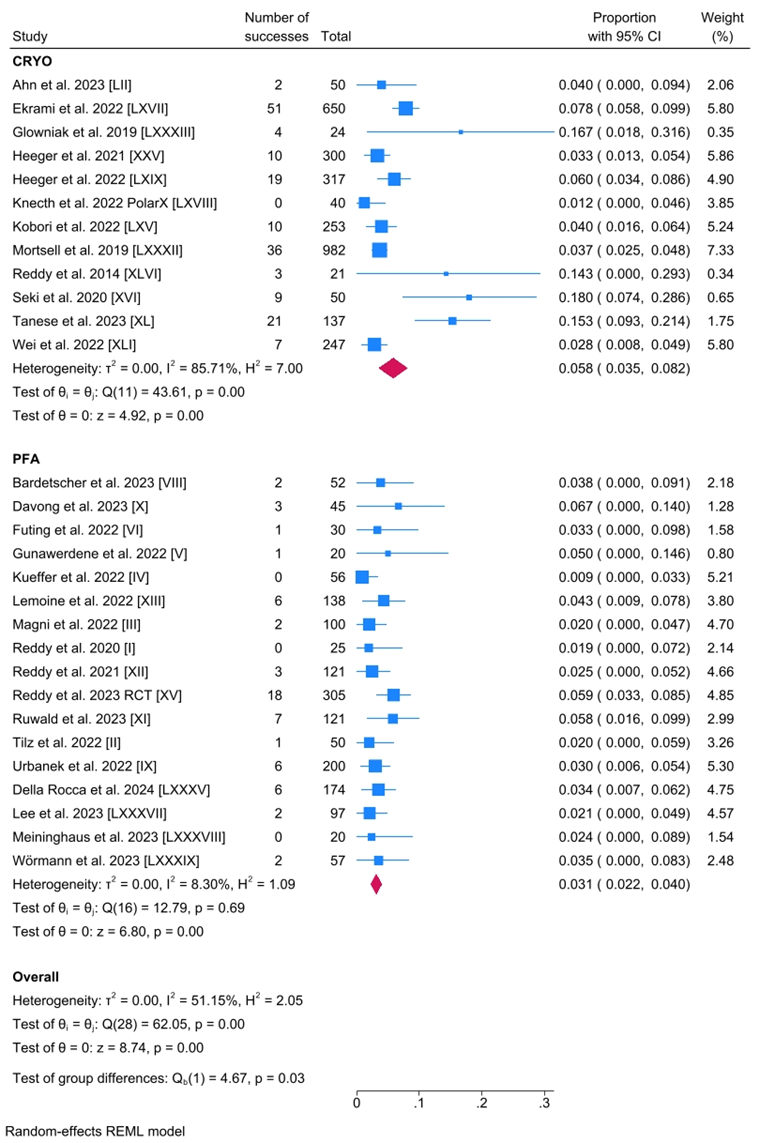
**

**Supplemental Figure 10. Forest plot comparing Major complications between Pulsed Field Ablation and Cryoballoon Ablation including only studies with 4^th^ generation cryoballoons.**

*
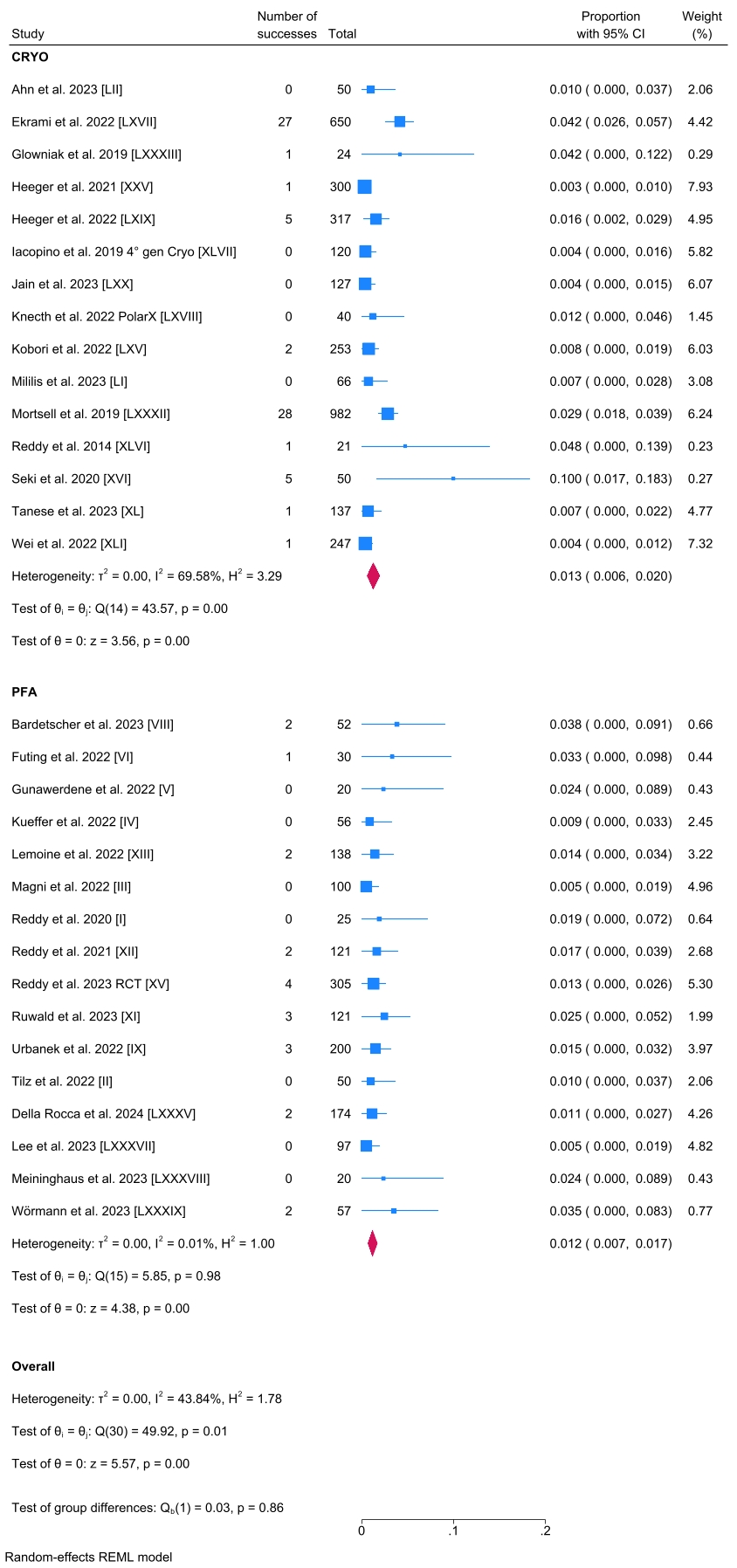
*

**Supplemental Figure 11. Forest plot comparing Procedural time (A) and Fluoroscopy time(B) between Pulsed Field Ablation and Cryoballoon Ablation including only studies with 4^th^ generation cryoballoons.**

**
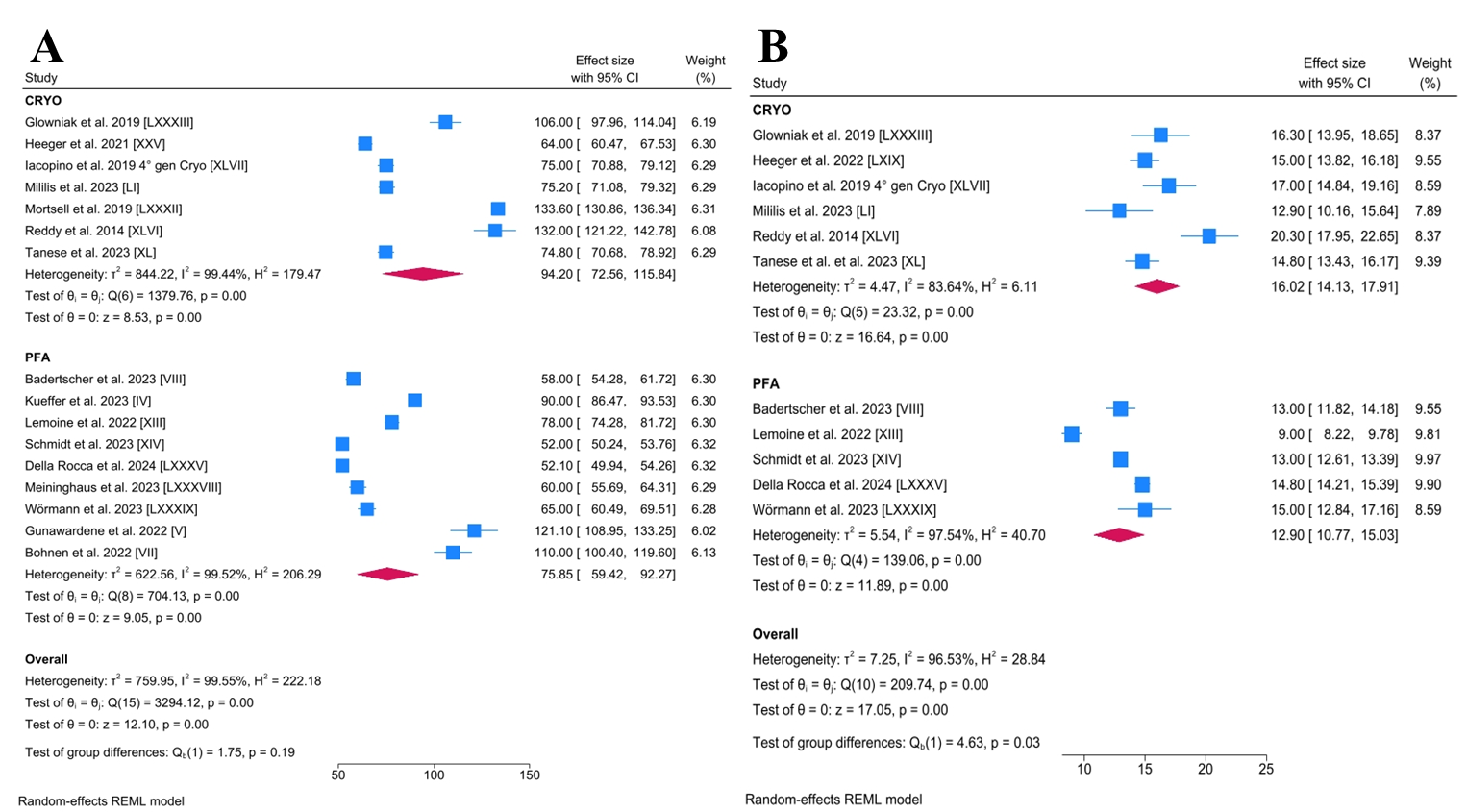
**

**Supplemental Figure 12: (A) Methodological quality graph and (B) methodological quality summary for the risk of bias from the included studies for Pulsed Field Ablation using the Newcastle–Ottawa Quality Assessment Scale for Cohort Studies tool.**

**
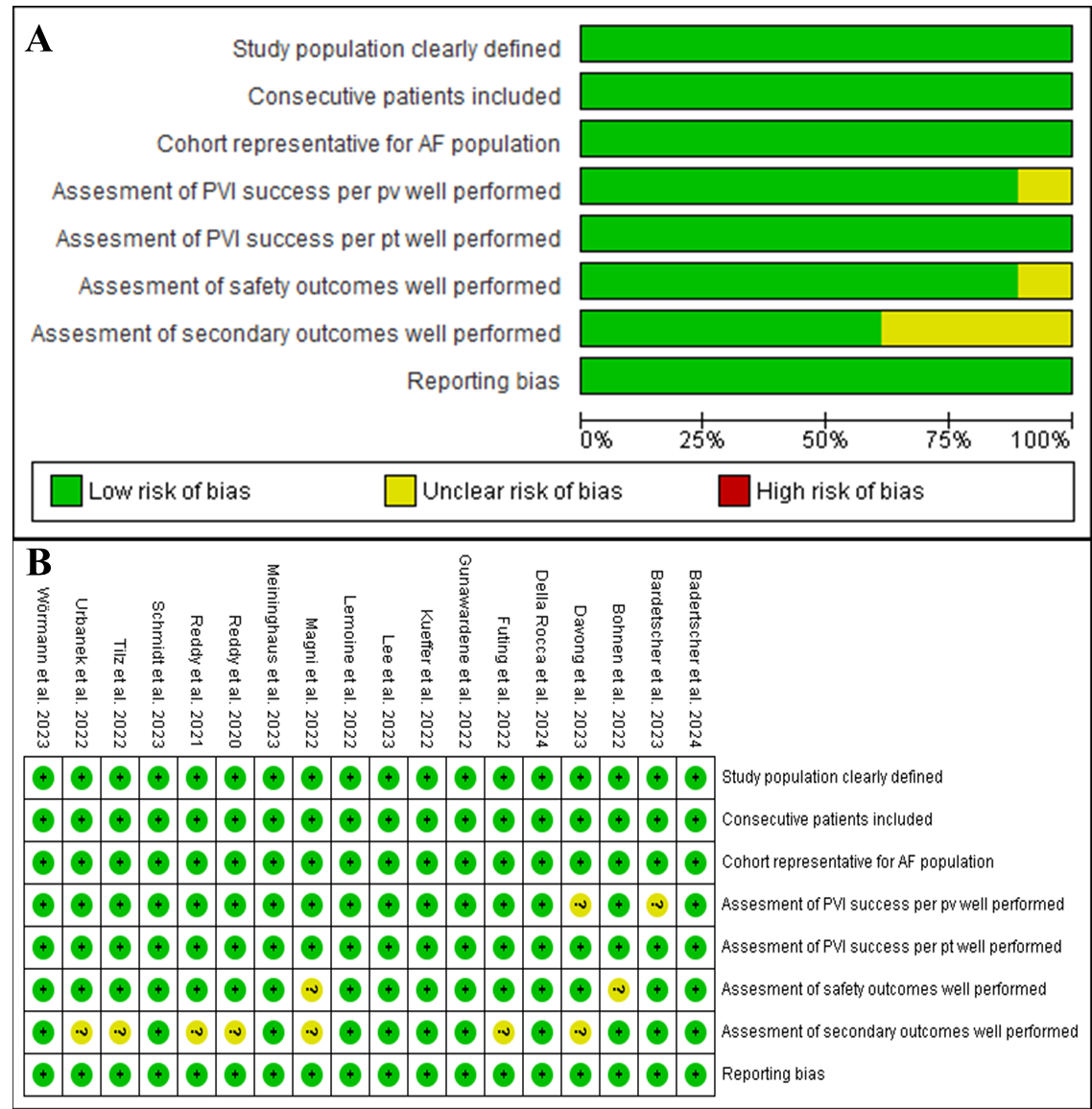
**

**Supplemental Figure 13: (A) Methodological quality graph and (B) methodological quality summary for the risk of bias from the included studies for Pulsed Field Ablation using Cochrane Risk of Bias tool for Randomized Controlled Trials.**

**
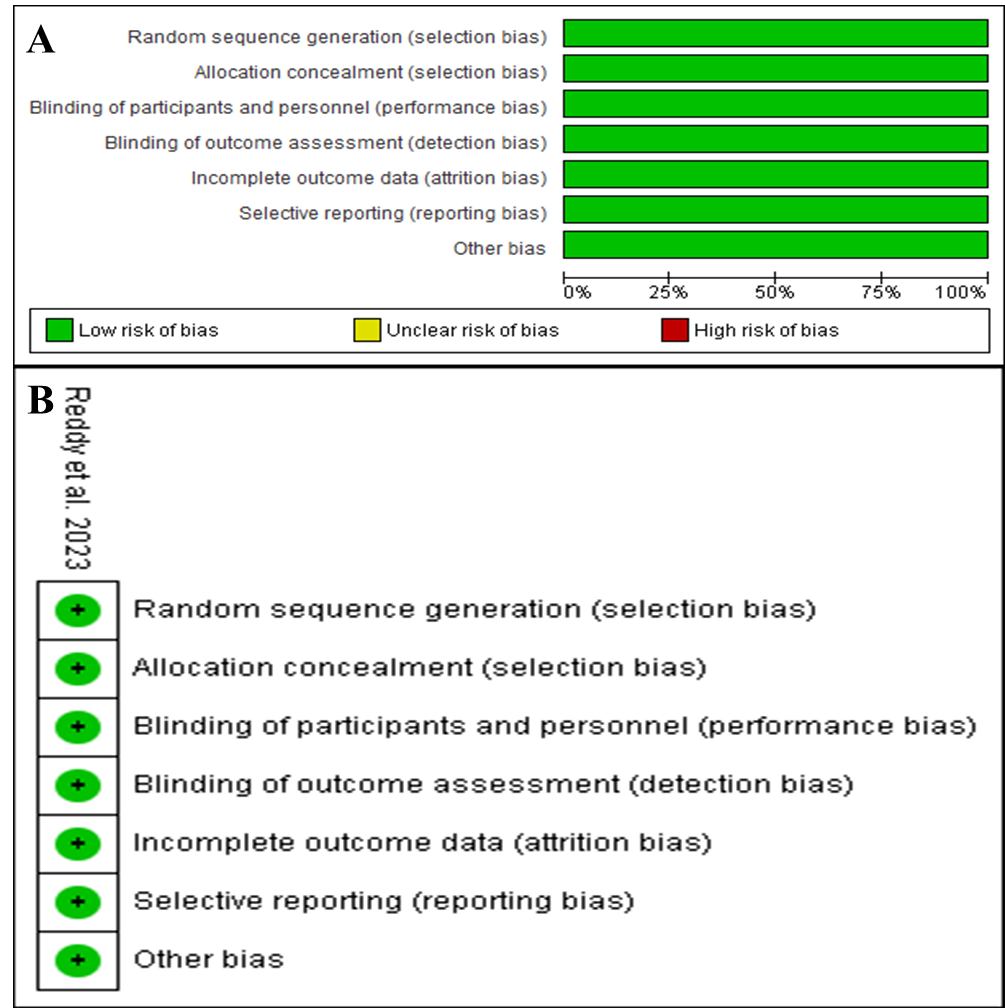
**

**Supplemental Figure 14: (A) Methodological quality graph and (B) methodological quality summary for the risk of bias from the included studies for Cryoballoon Ablation using the Newcastle–Ottawa Quality Assessment Scale for Cohort Studies tool.**


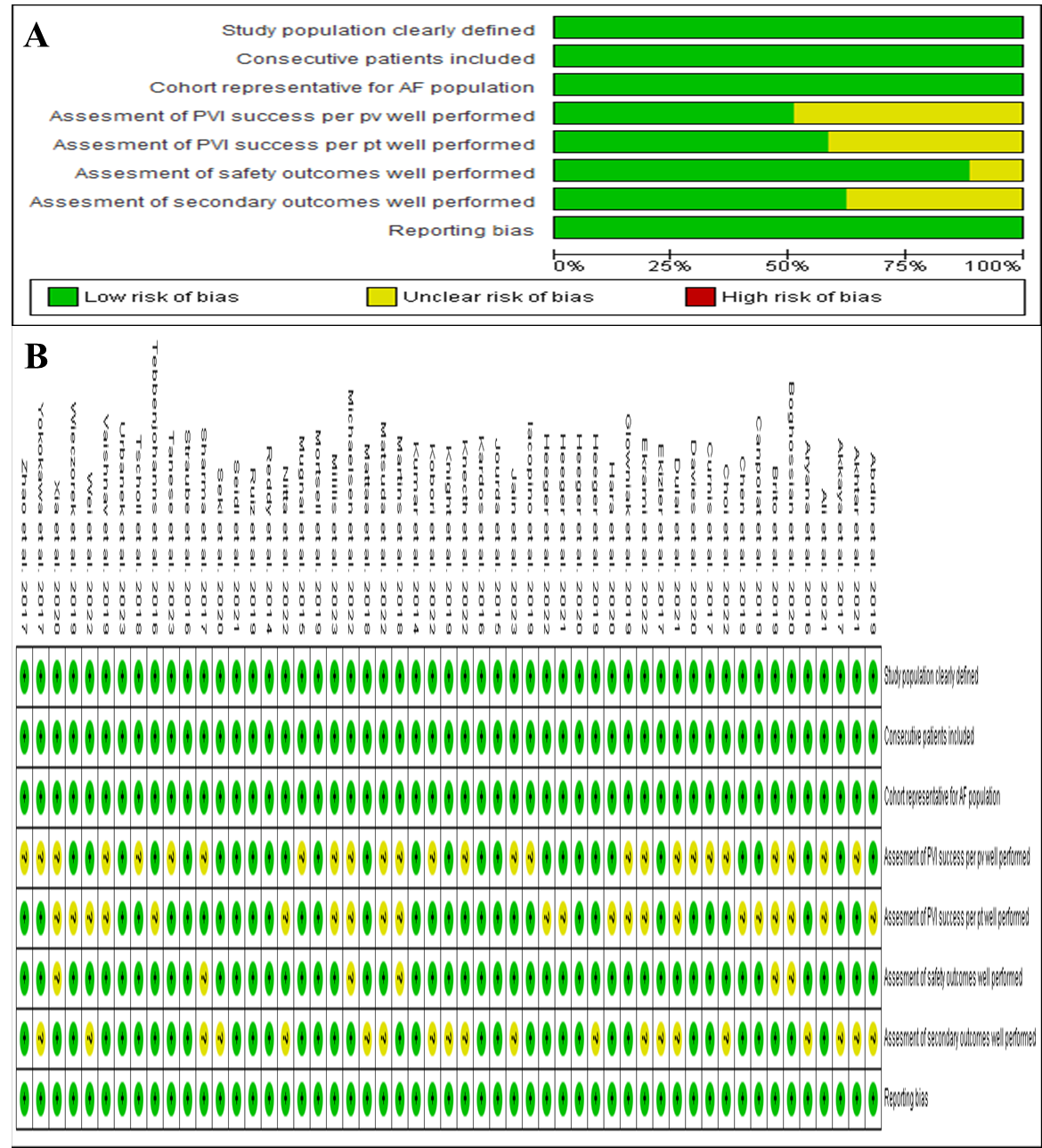


**Supplemental Figure 15: (A) Methodological quality graph and (B) methodological quality summary for the risk of bias from the included studies for Cryoballoon Ablation using Cochrane Risk of Bias tool for Randomized Controlled Trials.**

**
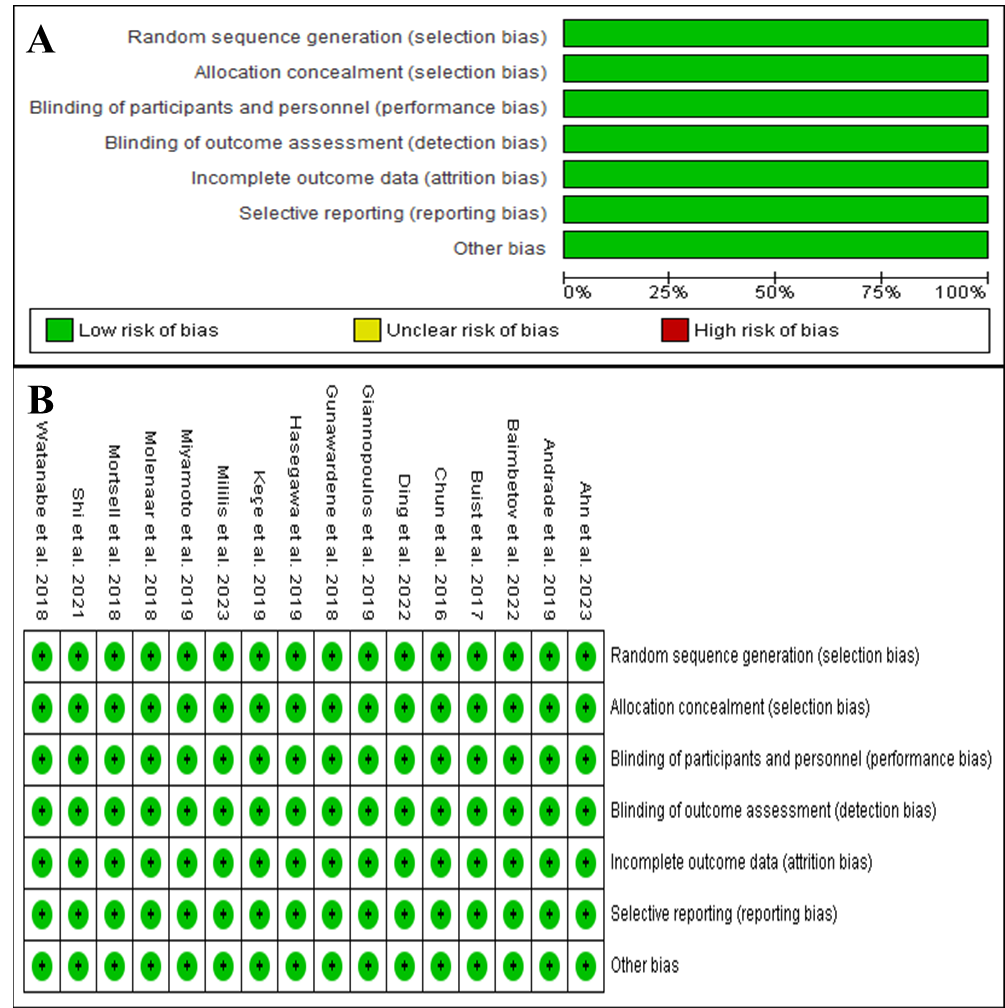
**

**Supplemental Figure 16: Funnel plot for visual inspection of publication bias for Acute Procedural Success per Vein (A), Acute Procedural Success per Patients (B), Overall Periprocedural Complications (C) and Major Periprocedural Complications (D).**


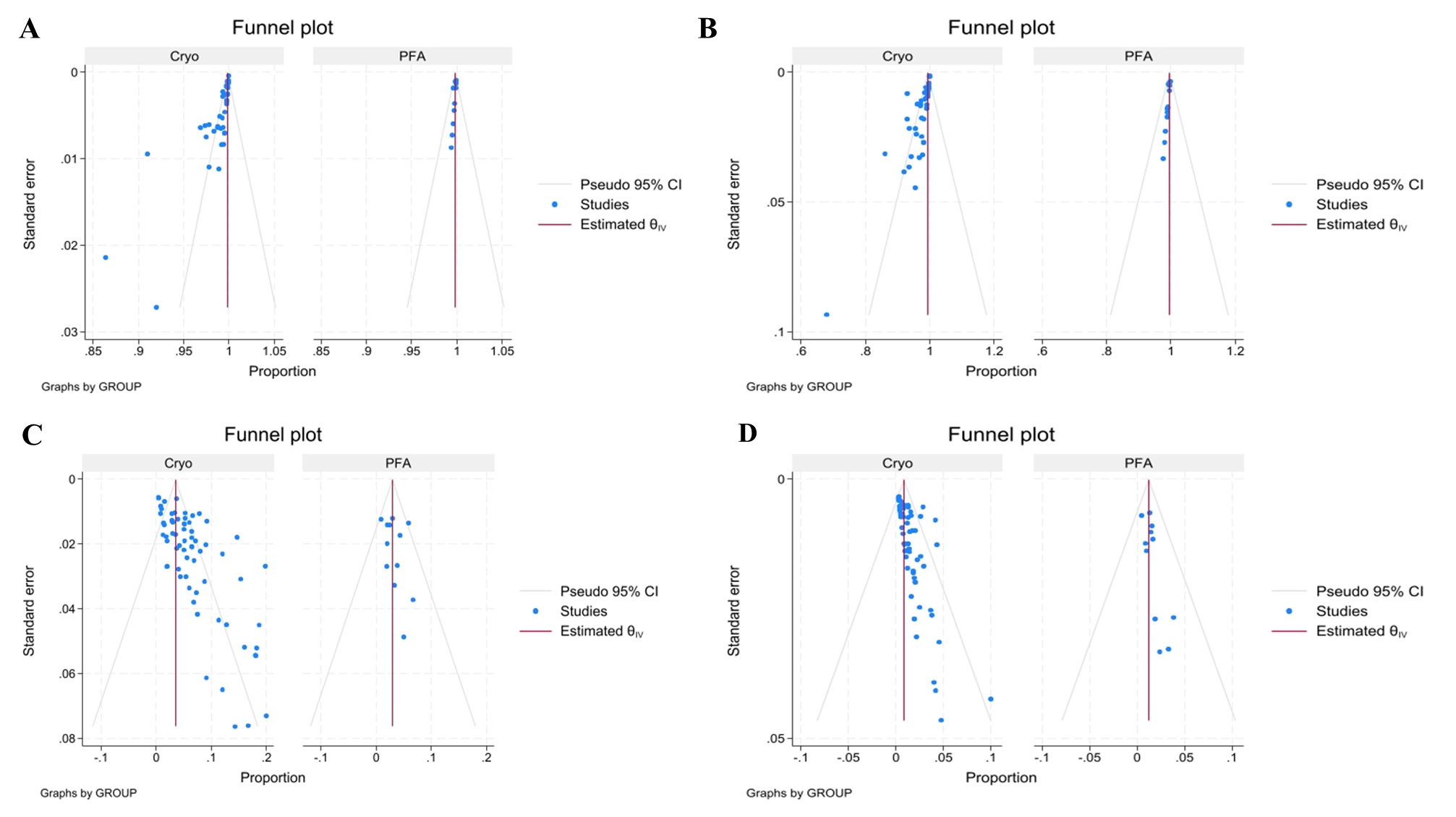


**References**

[I]. Reddy VY, Anic A, Koruth J, et al. Pulsed Field Ablation in Patients With Persistent Atrial Fibrillation. J Am Coll Cardiol. 2020;76:1068–1080.

[II]. Tilz RR, Vogler J, Kirstein B, et al. Pulsed Field Ablation-Based Pulmonary Vein Isolation Using a Simplified Single-Access Single-Catheter Approach　- The Fast and Furious PFA Study. Circ J. 2023. Published onlineAugust 3, 2023. https://doi.org/10.1253/circj.CJ-23-0389.

[III]. Magni FT, Mulder BA, Groenveld HF, et al. Initial experience with pulsed field ablation for atrial fibrillation. Front Cardiovasc Med. 2022;9:959186.

[IV]. Kueffer T, Stefanova A, Madaffari A, et al. Pulmonary vein isolation durability and lesion regression in patients with recurrent arrhythmia after pulsed-field ablation. J Interv Card Electrophysiol. 2023. Published onlineJuly 31, 2023. https://doi.org/10.1007/s10840-023-01608-7.

[V]. Gunawardene MA, Schaeffer BN, Jularic M, et al. Pulsed-field ablation combined with ultrahigh-density mapping in patients undergoing catheter ablation for atrial fibrillation: Practical and electrophysiological considerations. J Cardiovasc Electrophysiol. 2022;33:345–356.

[VI]. Füting A, Reinsch N, Höwel D, Brokkaar L, Rahe G, Neven K. First experience with pulsed field ablation as routine treatment for paroxysmal atrial fibrillation. Europace. 2022;24:1084–1092.

[VII]. Bohnen M, Weber R, Minners J, et al. Characterization of circumferential antral pulmonary vein isolation areas resulting from pulsed-field catheter ablation. Europace. 2023;25:65–73.

[VIII]. Badertscher P, Weidlich S, Serban T, et al. Pulsed-field ablation versus single-catheter high-power short-duration radiofrequency ablation for atrial fibrillation: Procedural characteristics, myocardial injury, and mid-term outcomes. Heart Rhythm. 2023:S1547-5271(23)02220–8.

[IX]. Urbanek L, Bordignon S, Schaack D, et al. Pulsed Field Versus Cryoballoon Pulmonary Vein Isolation for Atrial Fibrillation: Efficacy, Safety, and Long-Term Follow-Up in a 400-Patient Cohort. Circ Arrhythm Electrophysiol. 2023;16:389–398.

[X]. Davong B, Adeliño R, Delasnerie H, et al. Pulsed-Field Ablation on Mitral Isthmus in Persistent Atrial Fibrillation: Preliminary Data on Efficacy and Safety. JACC Clin Electrophysiol. 2023;9:1070–1081.

[XI]. Ruwald MH, Johannessen A, Hansen ML, Haugdal M, Worck R, Hansen J. Pulsed field ablation in real-world atrial fibrillation patients: clinical recurrence, operator learning curve and re-do procedural findings. J Interv Card Electrophysiol. 2023. Published onlineFebruary 8, 2023. https://doi.org/10.1007/s10840-023-01495-y.

[XII]. Reddy VY, Dukkipati SR, Neuzil P, et al. Pulsed Field Ablation of Paroxysmal Atrial Fibrillation: 1-Year Outcomes of IMPULSE, PEFCAT, and PEFCAT II. JACC Clin Electrophysiol. 2021;7:614–627.

[XIII]. Lemoine MD, Fink T, Mencke C, et al. Pulsed-field ablation-based pulmonary vein isolation: acute safety, efficacy and short-term follow-up in a multi-center real world scenario. Clin Res Cardiol. 2023;112:795–806.

[XIV]. Schmidt B, Bordignon S, Neven K, et al. EUropean real-world outcomes with Pulsed field ablatiOn in patients with symptomatic atRIAl fibrillation: lessons from the multi-centre EU-PORIA registry. Europace. 2023;25:euad185.

[XV]. Reddy VY, Gerstenfeld EP, Natale A, et al. Pulsed Field or Conventional Thermal Ablation for Paroxysmal Atrial Fibrillation. N Engl J Med. 2023. Published onlineAugust 27, 2023. https://doi.org/10.1056/NEJMoa2307291.

[XVI]. Seki R, Nagase T, Asano S, et al. Radiofrequency Current Versus Balloon-Based Ablation for Atrial Fibrillation. Am J Cardiol. 2022;178:52–59.

[XVII]. Curnis A, Salghetti F, Cerini M, et al. Efficacy of second-generation cryoballoon ablation in paroxysmal and persistent atrial fibrillation patients. J Cardiovasc Med (Hagerstown). 2017;18:655–662.

[XVIII]. Tokuda M, Yamashita S, Matsuo S, et al. Clinical significance of early recurrence of atrial fibrillation after cryoballoon vs. radiofrequency ablation-A propensity score matched analysis. PLoS One. 2019;14:e0219269.

[XIX]. Canpolat U, Kocyigit D, Yalcin MU, et al. Long-term outcomes of pulmonary vein isolation using second-generation cryoballoon during atrial fibrillation ablation. Pacing Clin Electrophysiol. 2019;42:910–921.

[XX]. Boghossian SHC, Barbosa EC, Boghossian E, et al. Experience in a Brazilian Center with Cryoablation for Electric Isolation of the Pulmonary Veins in Paroxysmal and Persistent Atrial Fibrillation - Preliminary Results in Brazil. Arq Bras Cardiol. 2020;115:528–535.

[XXI]. Brito V G, N V, L T, et al. Second Generation Cryoballoon vs. Radiofrequency Ablation in Paroxysmal Atrial Fibrillation: Outcomes Beyond One-Year Follow-up. J Atr Fibrillation. 2019;11:2147.

[XXII]. Zhao A, Squara F, Marijon E, Thomas O. Two-year clinical outcome after a single cryoballoon ablation procedure: A comparison of first- and second-generation cryoballoons. Arch Cardiovasc Dis. 2017;110:543–549.

[XXIII]. Akkaya E, Berkowitsch A, Zaltsberg S, et al. Second-generation cryoballoon ablation as a first-line treatment of symptomatic atrial fibrillation: Two-year outcome and predictors of recurrence after a single procedure. Int J Cardiol. 2018;259:76–81.

[XXIV]. Kumar N, Dinh T, Phan K, et al. Adenosine testing after second-generation cryoballoon ablation (ATSCA) study improves clinical success rate for atrial fibrillation. Europace. 2015;17:871–876.

[XXV]. Heeger C-H, Bohnen J-E, Popescu S, et al. Experience and procedural efficacy of pulmonary vein isolation using the fourth and second generation cryoballoon: The shorter, the better? J Cardiovasc Electrophysiol. 2021;32:1553–1560.

[XXVI]. Hara S, Miyazaki S, Hachiya H, et al. Long-term outcomes after second-generation cryoballoon ablation of paroxysmal atrial fibrillation - Feasibility of a single short freeze strategy without bonus applications. Int J Cardiol. 2020;306:90–94.

[XXVII]. Heeger C-H, Schuette C, Seitelberger V, et al. Time-to-effect guided pulmonary vein isolation utilizing the third-generation versus second generation cryoballoon: One year clinical success. Cardiol J. 2019;26:368–374.

[XXVIII]. Wieczorek M, Tajtaraghi S, Sassani K, Hoeltgen R. Incidence of early pulmonary vein reconnections using different energy sources for pulmonary vein isolation: Multielectrode phased radiofrequency vs second-generation cryoballoon. J Cardiovasc Electrophysiol. 2019;30:1428–1435.

[XXIX]. Yokokawa M, Chugh A, Latchamsetty R, et al. Ablation of paroxysmal atrial fibrillation using a second-generation cryoballoon catheter or contact-force sensing radiofrequency ablation catheter: A comparison of costs and long-term clinical outcomes. J Cardiovasc Electrophysiol. 2018;29:284–290.

[XXX]. Tebbenjohanns J, Höfer C, Bergmann L, et al. Shortening of freezing cycles provides equal outcome to standard ablation procedure using second-generation 28 mm cryoballoon after 15-month follow-up. Europace. 2016;18:206–210.

[XXXI]. Seidl P, Steinborn F, Costello-Boerrigter L, et al. Pulmonary vein isolation using second-generation single-shot devices: not all the same? J Interv Card Electrophysiol. 2021;60:521–528. [XXXII]. Jourda F, Providencia R, Marijon E, et al. Contact-force guided radiofrequency vs. second-generation balloon cryotherapy for pulmonary vein isolation in patients with paroxysmal atrial fibrillation-a prospective evaluation. Europace. 2015;17:225–231.

[XXXIII]. Straube F, Hartl S, Dorwarth U, et al. Cryoballoon ablation for persistent atrial fibrillation - Large single-center experience. J Cardiol. 2016;68:492–497.

[XXXIV]. Matta M, Anselmino M, Ferraris F, Scaglione M, Gaita F. Cryoballoon vs. radiofrequency contact force ablation for paroxysmal atrial fibrillation: a propensity score analysis. J Cardiovasc Med (Hagerstown). 2018;19:141–147.

[XXXV]. Ekizler A, Aras D, Cay S, Ozeke O, Ozcan F, Topaloglu S. Bonus vs No Bonus Cryoballoon Isolation for Paroxysmal Atrial Fibrillation Ablation. J Atr Fibrillation. 2017;9:1513.

[XXXVI]. Sharma A, Dhoot J, Wang J, Jones P, Gupta S, Wimmer AP. Outcomes following cryoballoon ablation for atrial fibrillation guided by pressure waveform monitoring without the routine use of pulmonary venography. J Interv Card Electrophysiol. 2017;49:75–82.

[XXXVII]. Glowniak A, Tarkowski A, Janczarek M, Wysokinski A. Silent cerebral infarcts following pulmonary vein isolation with different atrial fibrillation ablation techniques - incidence and risk factors. Arch Med Sci. 2022;18:632–638.

[XXXVIII]. Mugnai G, de Asmundis C, Ciconte G, et al. Incidence and characteristics of complications in the setting of second-generation cryoballoon ablation: A large single-center study of 500 consecutive patients. Heart Rhythm. 2015;12:1476–1482.

[XXXIX]. Nitta G, Nitta J, Inaba O, et al. Optimal single procedure strategy of pulmonary vein isolation with cryoballoon or radiofrequency and non-pulmonary vein triggers ablation for non-paroxysmal atrial fibrillation. Int J Cardiol Heart Vasc. 2022;40:101021.

[XL]. Tanese N, Almorad A, Pannone L, et al. Outcomes after cryoballoon ablation of paroxysmal atrial fibrillation with the PolarX or the Arctic Front Advance Pro: a prospective multicentre experience. Europace. 2023;25:873–879.

[XLI]. Wei Y, Chen L, Cao J, et al. Long-term outcomes of a time to isolation - based strategy for cryoballoon ablation compared to radiofrequency ablation in patients with symptomatic paroxysmal atrial fibrillation. Pacing Clin Electrophysiol. 2022;45:1015–1023.

[XLII]. Michaelsen J, Parade U, Bauerle H, et al. Twelve-month efficacy of second-generation cryoballoon ablation for atrial fibrillation performed at community hospitals: results of the German register on cryoballoon ablation in local hospitals (regional). J Interv Card Electrophysiol. 2023;66:417–425.

[XLIII]. Knight BP, Novak PG, Sangrigoli R, et al. Long-Term Outcomes After Ablation for Paroxysmal Atrial Fibrillation Using the Second-Generation Cryoballoon: Final Results From STOP AF Post-Approval Study. JACC Clin Electrophysiol. 2019;5:306–314.

[XLIV]. Ali AN, Riad O, Tawfik M, Opel A, Wong T. Newer generation cryoballoon vs. contact force-sensing radiofrequency ablation catheter in the ablation of paroxysmal atrial fibrillation. Herzschrittmacherther Elektrophysiol. 2021;32:236–243.

[XLV]. Aryana A, Kowalski M, O’Neill PG, et al. Catheter ablation using the third-generation cryoballoon provides an enhanced ability to assess time to pulmonary vein isolation facilitating the ablation strategy: Short- and long-term results of a multicenter study. Heart Rhythm. 2016;13:2306–2313.

[XLVI]. Reddy VY, Sediva L, Petru J, et al. Durability of Pulmonary Vein Isolation with Cryoballoon Ablation: Results from the Sustained PV Isolation with Arctic Front Advance (SUPIR) Study. J Cardiovasc Electrophysiol. 2015;26:493–500.

[XLVII]. Iacopino S, Pieragnoli P, Arena G, et al. A comparison of acute procedural outcomes within four generations of cryoballoon catheters utilized in the real-world multicenter experience of 1STOP. J Cardiovasc Electrophysiol. 2020;31:80–88.

[XLVIII]. Baimbetov A, Bizhanov K, Yakupova I, et al. Efficacy and Safety Results of Different Ablation Technologies for Persistent Atrial Fibrillation Treatment. Heart Surg Forum. 2022;25:E594–E600.

[XLIX]. Ding J, Cheng A, Li P, et al. Cryoballoon catheter ablation or drug therapy to delay progression of atrial fibrillation: A single-center randomized trial. Front Cardiovasc Med. 2022;9:1003305.

[L]. Shi L-B, Rossvoll O, Tande P, Schuster P, Solheim E, Chen J. Cryoballoon vs. radiofrequency catheter ablation: insights from NOrwegian randomized study of PERSistent Atrial Fibrillation (NO-PERSAF study). Europace. 2022;24:226–233.

[LI]. Mililis P, Kariki O, Saplaouras A, et al. Radiofrequency versus cryoballoon catheter ablation in patients with persistent atrial fibrillation: A randomized trial. J Cardiovasc Electrophysiol. 2023;34:1523–1528.

[LII]. Ahn J, Shin DG, Han S-J, Lim HE. Safety and efficacy of intracardiac echocardiography-guided zero-fluoroscopic cryoballoon ablation for atrial fibrillation: a prospective randomized controlled trial. Europace. 2023;25:euad086.

[LIII]. Gunawardene MA, Hoffmann BA, Schaeffer B, et al. Influence of energy source on early atrial fibrillation recurrences: a comparison of cryoballoon vs. radiofrequency current energy ablation with the endpoint of unexcitability in pulmonary vein isolation. Europace. 2018;20:43–49.

[LIV]. Buist TJ, Adiyaman A, Smit JJJ, Ramdat Misier AR, Elvan A. Arrhythmia-free survival and pulmonary vein reconnection patterns after second-generation cryoballoon and contact-force radiofrequency pulmonary vein isolation. Clin Res Cardiol. 2018;107:498–506.

[LV]. Hasegawa K, Miyazaki S, Kaseno K, et al. Pressure-guided second-generation cryoballoon pulmonary vein isolation: Prospective comparison of the procedural and clinical outcomes with the conventional strategy. J Cardiovasc Electrophysiol. 2019;30:1841–1847.

[LVI]. Mörtsell D, Malmborg H, Lönnerholm S, Jansson V, Blomström-Lundqvist C. Acute and long-term efficacy and safety with a single cryoballoon application as compared with the standard dual application strategy: a prospective randomized study using the second-generation cryoballoon for pulmonary vein isolation in patients with symptomatic atrial fibrillation. Europace. 2018;20:1598–1605.

[LVII]. Keçe F, de Riva M, Naruse Y, et al. Optimizing ablation duration using dormant conduction to reveal incomplete isolation with the second generation cryoballoon: A randomized controlled trial. J Cardiovasc Electrophysiol. 2019;30:902–909.

[LVIII]. Molenaar MMD, Timmermans CC, Hesselink T, et al. Shorter cryoballoon applications times do effect efficacy but result in less phrenic nerve injury: Results of the randomized 123 study. Pacing Clin Electrophysiol. 2019;42:508–514.

[LIX]. Chun KRJ, Stich M, Fürnkranz A, et al. Individualized cryoballoon energy pulmonary vein isolation guided by real-time pulmonary vein recordings, the randomized ICE-T trial. Heart Rhythm. 2017;14:495–500.

[LX]. Giannopoulos G, Kossyvakis C, Vrachatis D, et al. Effect of cryoballoon and radiofrequency ablation for pulmonary vein isolation on left atrial function in patients with nonvalvular paroxysmal atrial fibrillation: A prospective randomized study (Cryo-LAEF study). J Cardiovasc Electrophysiol. 2019;30:991–998.

[LXI]. Andrade JG, Champagne J, Dubuc M, et al. Cryoballoon or Radiofrequency Ablation for Atrial Fibrillation Assessed by Continuous Monitoring: A Randomized Clinical Trial. Circulation. 2019;140:1779–1788.

[LXII]. Watanabe R, Sairaku A, Yoshida Y, et al. Head-to-head comparison of acute and chronic pulmonary vein stenosis for cryoballoon versus radiofrequency ablation. Pacing Clin Electrophysiol. 2018;41:376–382.

[LXIII]. Akhtar T, Wallace R, Daimee UA, et al. Safety and efficacy of cryoballoon versus radiofrequency ablation for atrial fibrillation in elderly patients: A real-world evidence. Indian Pacing Electrophysiol J. 2022;22:24–29.

[LXIV]. Matsuda Y, Masuda M, Sakio T, et al. Safety, efficacy, and cost-performance of a simplified cryoballoon ablation procedure for paroxysmal atrial fibrillation. J Interv Card Electrophysiol. 2022;64:427–435.

[LXV]. Kobori A, Sasaki Y, Pak M, et al. Comparison of Cryoballoon and Contact Force-Sensing Radiofrequency Ablation for Persistent Atrial Fibrillation in Clinical Practice. Circ J. 2022;86:290–298.

[LXVI]. Choi J-H, Park S-J, Park K-M, Kim JS, On YK. Efficacy and safety of cryoballoon pulmonary vein isolation for paroxysmal and persistent atrial fibrillation: A comparison with radiofrequency ablation. PLoS One. 2022;17:e0265482.

[LXVII]. Ekrami NK, Magni FT, Dayalani V, et al. Assessment of sex-related differences and outcome in patients who underwent cryoballoon pulmonary vein isolation for atrial fibrillation: an observational cohort study. BMJ Open. 2022;12:e063359.

[LXVIII]. Knecht S, Sticherling C, Roten L, et al. Efficacy and safety of a novel cryoballoon ablation system: multicentre comparison of 1-year outcome. Europace. 2022;24:1926–1932.

[LXIX]. Heeger C-H, Pott A, Sohns C, et al. Novel cryoballoon ablation system for pulmonary vein isolation: multicenter assessment of efficacy and safety-ANTARCTICA study. Europace. 2022;24:1917–1925.

[LXX]. Jain A, Chen C-C, Chang S-L, et al. Comparison of efficacy of pulmonary vein isolation between cryoballoon ablation and high-power short-duration ablation. Indian Pacing Electrophysiol J. 2023;23:110–115.

[LXXI]. Vaishnav AS, Levine E, Coleman KM, et al. Early recurrence of atrial fibrillation after pulmonary vein isolation: a comparative analysis between cryogenic and contact force radiofrequency ablation. J Interv Card Electrophysiol. 2020;57:67–75.

[LXXII]. Xia Y, Liu J, Jia Y, et al. Redefining the Blanking Period by a Long-Term Follow-Up after Atrial Fibrillation Ablation Using Second-Generation Cryoballoon. Int Heart J. 2020;61:936–943.

[LXXIII]. Heeger C-H, Subin B, Wissner E, et al. Second-generation cryoballoon-based pulmonary vein isolation: Lessons from a five-year follow-up. Int J Cardiol. 2020;312:73–80.

[LXXIV]. Davies A, Mahmoodi E, Emami M, et al. Comparison of Outcomes Using the First and Second Generation Cryoballoon to Treat Atrial Fibrillation. Heart Lung Circ. 2020;29:452–459.

[LXXV]. Martins RP, Galand V, Cesari O, et al. The second generation cryoballoon has improved durable isolation of left but not right pulmonary veins: new insights from a multicentre study. Europace. 2018;20:1115–1121.

[LXXVI]. Chen C-F, Liu M-J, Jin C-L, Gao X-F, Liu X-H, Xu Y-Z. Costs and long-term outcomes following pulmonary vein isolation for atrial fibrillation in elderly patients using second-generation cryoballoon vs. open-irrigated radiofrequency in China. J Interv Card Electrophysiol. 2020;59:557–564.

[LXXVII]. Tscholl V, Lin T, Lsharaf AK-A, et al. Cryoballoon ablation in the elderly: one year outcome and safety of the second-generation 28mm cryoballoon in patients over 75 years old. Europace. 2018;20:772–777.

[LXXVIII]. Elvira Ruiz G, Peñafiel Verdú P, Martínez Sánchez J, et al. Medium- and long-term outcome after a single second-generation cryoballoon ablation in patients with paroxysmal and persistent atrial fibrillation. Rev Port Cardiol (Engl Ed). 2019;38:839–843.

[LXXIX]. Kardos A, Kis Z, Som Z, Nagy Z, Foldesi C. Two-Year Follow-Up after Contact Force Sensing Radiofrequency Catheter and Second-Generation Cryoballoon Ablation for Paroxysmal Atrial Fibrillation: A Comparative Single Centre Study. Biomed Res Int. 2016;2016:6495753.

[LXXX]. Dulai R, Uy CP, Kassir Y, et al. The long-term effect of thermal-guided second-generation cryoablation in paroxysmal and persistent atrial fibrillation. Indian Pacing Electrophysiol J. 2021;21:261–266.

[LXXXI]. Abdin A, Yalin K, Lyan E, et al. Safety and efficacy of cryoballoon ablation for the treatment of atrial fibrillation in elderly patients. Clin Res Cardiol. 2019;108:167–174.

[LXXXII]. Mörtsell D, Arbelo E, Dagres N, et al. Cryoballoon vs. radiofrequency ablation for atrial fibrillation: a study of outcome and safety based on the ESC-EHRA atrial fibrillation ablation long-term registry and the Swedish catheter ablation registry. Europace. 2019;21:581–589.

[LXXXIII] Glowniak A, Tarkowski A, Janczarek M, Wysokinski A. Silent cerebral infarcts following pulmonary vein isolation with different atrial fibrillation ablation techniques - incidence and risk factors. Arch Med Sci AMS. 2022;18:632–638.

[LXXXIV] Miyamoto K, Doi A, Hasegawa K, Morita Y, Mishima T, Suzuki I, Kaseno K, Nakajima K, Kataoka N, Kamakura T, Wada M, Yamagata K, Ishibashi K, Inoue YY, Nagase S, Noda T, Aiba T, Asakura M, Izumi C, Noguchi T, Tada H, Takagi M, Yasuda S, Kusano KF. Multicenter Study of the Validity of Additional Freeze Cycles for Cryoballoon Ablation in Patients With Paroxysmal Atrial Fibrillation: The AD-Balloon Study. Circ Arrhythm Electrophysiol. 2019;12:e006989.

[LXXXV] Della Rocca DG, Marcon L, Magnocavallo M, et al. Pulsed Electric Field, Cryoballoon, and Radiofrequency for Paroxysmal Atrial Fibrillation Ablation: A Propensity Score-Matched Comparison. *Europace*. 2024:euae016.

[LXXXVI] Badertscher P, Mannhart D, Weidlich S, et al. Left atrial posterior wall isolation using pulsed-field ablation: procedural characteristics, safety, and mid-term outcomes. *J Interv Card Electrophysiol*. 2024. Published onlineJanuary 5, 2024. https://doi.org/10.1007/s10840-023-01728-0.

[LXXXVII] Lee XW, Freeman BM, Gunthorpe NG, et al. Pulsed Field Ablation of Atrial Fibrillation: An Initial Australian Single-Centre Experience. *Heart Lung Circ*. 2023:S1443-9506(23)04405–0.

[LXXXVIII] Grosse Meininghaus D, Freund R, Koerber B, Kleemann T, Matthes H, Geller JC. Pulsed-field ablation does not induce esophageal and periesophageal injury-A new esophageal safety paradigm in catheter ablation of atrial fibrillation. *J Cardiovasc Electrophysiol*. 2024;35:86–93.

[LXXXIX] Wörmann J, Schipper J-H, Lüker J, et al. Comparison of pulsed-field ablation versus very high power short duration-ablation for pulmonary vein isolation. *J Cardiovasc Electrophysiol*. 2023;34:2417–2424.

[XC] Maurhofer J, Kueffer T, Madaffari A, et al. Pulsed-field vs. cryoballoon vs. radiofrequency ablation: a propensity score matched comparison of one-year outcomes after pulmonary vein isolation in patients with paroxysmal atrial fibrillation. *J Interv Card Electrophysiol*. 2023. Published onlineSeptember 30, 2023. https://doi.org/10.1007/s10840-023-01651-4.
